# Supplementary material for: Scrutinizing the protein hydration shell from molecular dynamics simulations against consensus small-angle scattering data
Source: Commun Chem. 2023 Dec 12;6:272. doi: 10.1038/s42004-023-01067-1 (PMC10716392; doi:10.1038/s42004-023-01067-1)
Supplement: Supplementary file 2 — Supplementary Information [file 42004_2023_1067_MOESM2_ESM.pdf]

Supplementary material for: Scrutinizing the protein hydration shell from molecular dynamics simulations against consensus small-angle scattering data

Johanna-Barbara Linse and Jochen S. Hub*

Theoretical Physics and Center for Biophysics, Saarland University, Saarbrücken, 66123, Germany

E-mail: jochen.hub@uni-saarland.de

Supplementary Material Table of Contents

Supplementary Discussion

Choice of SAS data used for quantitative comparison with MD simulations 3
Validation with R_g from $P(r)$ vs. Guinier analysis 4

Supplementary Results: additional control SAS calculations

Counter ion cloud contributions outside the envelope are negligible 4
Effect of salt relative to using only counter ions 5
Effect of cutoff settings for Lennard-Jones interactions 6
Effect of refined Lennard-Jones parameters to avoid Na^+–carboxylate overbinding in AMBER simulations (CUFIX) 6
Density correction for accurate buffer matching is critical for R_g predictions 7

Supplementary Methods

Solvent density profiles around restrained bulk water 8

Supplementary Figures

S1	Simulation system of xylanase	10
S2	Visual representations of five proteins	11
S3	Schematic representation of hydration shell contrast for SAXS, SANS/H_2O, SANS/D_2O and effect on ΔR_g	12
S4	Effect of protein flexibility	13
S5	Absolute R_g values of five proteins	14
S6	ΔR_g^{SAS} values from simulations with position restraints on backbone atoms	15
S7	Solvent density vs. distance from protein for three force fields	16
S8	Solvent density modulations around protein and restrained water	17
S9	RNaseA structures from Amber99SBws–OPC simulations	18
S10	ΔR_g^{SAS} for lysozyme and urate oxidase	19
S11	ΔR_g^{SAS} compared to experimental R_g values from Guinier analysis	20
S12	Effects of salt vs. only counter ions for xylanase	21
S13	Effects of salt vs. only counter ions for RNaseA	22
S14	Effects of salt vs. only counter ions for glucose isomerase	23
S15	ΔR_g^{SAS} values for glucose isomerase from simulations using only counter ions for 18 force field combinations	24
S16	Modified cation–carboxylate Lennard-Jones parameters with Amber force fields (CUFIX)	25
S17	Force-switch vs. plain cutoff with CHARMM36m	26

Supplementary Tables

S1	List of force fields and water models considered in this study	27
S2	Experimental consensus R_g values from Trewhella et al.[1]	28
S3	All computed ΔR_g values of RNaseA	29
S4	All computed ΔR_g values of xylanase	30
S5	All computed ΔR_g values of glucose isomerase	31
S6	All computed ΔR_g values of lysozyme	32
S7	All computed ΔR_g values of urate oxidase	33

Supplementary Discussion

Choice of SAS data used for quantitative comparison with MD simulations

Trewhella, Vachette, et al. recently reported a worldwide round-robin benchmark on the reproducibility of SAXS and SANS experiments, involving experiments at 12 SAXS and four SANS instruments and leading to a total of 171 SAXS and 76 SANS curves on the following five proteins (Fig. S2): ribonuclease A (RNaseA, 13.690 kDa), hen egg white lysozyme (14.313 kDa), xylanase (20.844 kDa), urate oxidase (136.303 kDa), and glucose isomerase (172.910 kDa, also referred to as xylose isomerase).[1] Both, SAXS and SANS data were collected in batch mode or coupled to size-exclusion chromatography (SEC-SAXS, SEC-SANS). The R_g values were evaluated using Guinier analysis and based on the $P(r)$ function. Generally, good agreement was found between the experimental SAS curves and explicit-solvent SAXS predictions in the round-robin study.[1]

However, the quality and reproducibility of the SAS data differed among the five proteins. Specifically, R_g values for lysozyme varied substantially between different data sets, even if experiments were carried out in SEC mode. These variations have been attributed to an increased sensitivity of lysozyme to radiation damage[1] and are in line with variations of the lysozyme R_g in earlier reports.[2,3]

Urate oxidase exhibits increased propensity for aggregation and, thus, had to be shipped on ice and with a reduced concentration of only 5 mg ml^{-1}.[1] In consequence, SAS data of urate oxidate was subject to poorer statistics as compared to the data of the other four proteins, in particular at wider scattering angles. Because the SAS curve over the whole q-range is used when computing R_g via the $P(r)$ function, such problems may lead to increased uncertainties of the R_g estimate. Furthermore, SANS in H_2O exhibited exceptionally high background, and yielded unrealistic R_g values from Guinier analysis. Owing to these problems with lysozyme and urate oxidase, we did not include these proteins in the quantitative comparison of ΔR_g^{SAS} values in Fig. 3. For the sake of completeness, the R_g^{SAS} values

for lysozyme and urate oxidase, together with the experimental R_g estimates from Guinier analysis and $P(r)$, are shown in Fig. S10.

Validation with R_g from $P(r)$ vs. Guinier analysis

In this study, we used the experimental R_g values obtained from the $P(r)$ function and not from the Guinier analysis for comparison with MD simulations. In contrast to the Guinier analysis that uses only the small-angle region of the SAS curve, the $P(r)$ function makes use of the entire SAS curve. Thus, R_g values from the $P(r)$ reported in the round-robin SAS benchmark study were less sensitive to remaining undetected protein–protein aggregation and subject to smaller statistical uncertainties as compared to R_g estimates from the Guinier analysis (Table S2).[1] Nevertheless, the $\Delta R_\mathrm{g}^\mathrm{SAS}$ values from simulations reveal reasonable agreement also with R_g from Guinier analysis (Fig. S11).

Supplementary Results: additional control SAS calculations

Counter ion cloud contributions outside the envelope are negligible

For the highly anionic glucose isomerase (GI), the counter ion cloud adds to the contrast of the hydration layer. According to Debye-Hückel theory, the counter ion cloud decays with the Debye length λ_D into the bulk solvent, which equals $\lambda_D = 9.7\,\mathrm{Å}$ for a 100 mM NaCl solution. Hence, since only solvent atoms within the envelope at a distance of 9 Å from the protein (Fig. 1a, blue surface) contributed to our explicit-solvent SAS calculations, counter ion cloud effects were likewise only taken into account up to a distance of 9Å from the protein surface, whereas counter ion cloud contributions beyond 9Å were neglected.

To exclude that this approximation affects the computed SAS curves, we estimated the effect of the counter ion cloud at distances beyond 9 Å on R_g. Following Ref. 4, we modeled GI as a sphere with contrast, volume, and R_g taken from GI, and we used linearized Poisson–Boltzmann calculations to obtain the number densities of sodium and chloride as function of distance R from the sphere surface. By computing R_g including the counter ion

cloud up to $R = 9\,\text{Å}$ or up to a large distance, we found that R_g was modified by the cutoff at $R = 9\,\text{Å}$ by only $0.033\,\text{Å}$, $-0.062\,\text{Å}$ and $-0.03\,\text{Å}$ for SAXS, SANS in H_2O, and SANS in D_2O. Since these errors are within the statistical errors, these effects were neglected for further analysis.

Effect of salt relative to using only counter ions

Since GI carries a large negative charge ($-60\,e$), GI is surrounded by a counter ion cloud. The ion densities in the counter ion cloud decay into the bulk solvent with the Debye length λ_D, which is inversely proportional to the square root of the ion concentration in bulk solvent.[5] Thus, upon simulating with a smaller ion concentration relative to experimental conditions, the spatial extend of the counter ion cloud in simulation would exceed the experimental conditions; consequently, the counter ion cloud in simulations would, relative to the experiment, impose an electron density contrast at larger distances from the protein, which may lead to an overestimated R_g. We tested the effect of the ion concentration for GI by computing R_g, ΔR_g, and $\Delta R_\text{g}^\text{SAS}$ values from simulations with only counter ions, 100 mM NaCl salt, or 150 mM NaCl salt (Fig. S14). We find that, with increasing salt concentration, $\Delta R_\text{g}^\text{SAS}$ values decrease, as expected from a deceasing Debye length and, thereby, from a spatially more compact counter ion cloud. For instance, in simulations with CHARMM36m–TIP3P, upon replacing counter ions with 150 mM NaCl salt, the R_g from SAXS relative to SANS/D_2O decreases by ~0.2Å. This trend is confirmed by GI simulations with all 18 force field combinations using purely counter ions (compare Fig. S15 with Fig. 3e/f). Thus, for quantitative comparison of $\Delta R_\text{g}^\text{SAS}$ values between simulation and experiment, an at least approximate match of the buffer conditions is mandatory, as used for our study.

In addition, we tested the effect of adding additional 150 mM NaCl to simulation of xylanase and RNaseA instead of using only counter ions (Figs. S12 and S13). For these near-neutral proteins, $\Delta R_\text{g}^\text{SAS}$ values agreed within error bars between simulations with salt and simulations with counter ions, as expected form the absence of a pronounced counter

ion cloud.

Effect of cutoff settings for Lennard-Jones interactions

Neglect of Lennard-Jones (LJ) interactions beyond the cutoff could in principle modify the density of the hydration shell. Thus, we tested the effects of LJ cutoffs for xylanase simulated with the CHARMM36m–TIP3P force field combination. The CHARMM36m force field has been parameterized with a gradual switch of LJ forces between 1 and 1.2 nm. We carried out xylanase simulations either (i) following the CHARMM recommendations (force-switch) or (ii) using a plain cutoff at 1 nm. For each cutoff setting, simulations were carried out with restraints on heavy atoms, restraints on the backbone, or using a unrestrained MD simulation (Fig. S17). For simulations with restraints on the backbone or for unrestrained MD simulations, we obtained larger ΔR_g^{SAS} values with the force-switch settings as compared to using a plain cutoff (Fig. S17, orange vs. yellow and lightblue vs. darkblue bars). Hence, upon validating force fields against ΔR_g^{SAS} values from experiments, it is critical to follow the cutoff settings that have been used during force field parametrization.

Notably, for simulations with restraints on heavy atoms, no such effect was visible (Fig. S17, pink vs. red bars). Thus, additional LJ interactions between 1 nm and 1.2 nm lead to larger ΔR_g^{SAS} only in the presence of amino acid side chain fluctuations. These findings demonstrate that the additional LJ interactions between 1 nm and 1.2 nm with force-switch settings lead to increased ΔR_g^{SAS} values not because of tighter packing of water on a given protein surface; instead, the additional interactions lead to more favorable protein–water conformations, which manifest in a larger density contrast of the hydration shell.

Effect of refined Lennard-Jones parameters to avoid Na^+–carboxylate overbinding in AMBER simulations (CUFIX)

Certain older models of monovalent cations such as Na^+ tend to overbind to carboxylate moieties, as present in aspartate and glutamate residues.[6,7] We found previously that such

overbinding of monovalent ions to anionic proteins may influence the R_g values obtained from explicit-solvent SAS calculations.[8] Whereas refined LJ parameters have been incorporated into the default CHARMM36m force field to avoid overbinding (denoted NBFIX or CUFIX), refined LJ parameters for AMBER force fields have become available only recently.[7]

As discussed in the Results, ΔR_g^{SAS} values for GI obtained with CHARMM36m were systematically larger as compared to values obtained with AMBER force fields, irrespective of the applied water model (Fig. 3e/f). We hypothesized that different binding properties of Na^+ ions in CHARMM36m as compared to AMBER simulations may partly explain such effect. To test this hypothesis, we carried out additional SAS calculations for GI with the ff14SB force field with CUFIX and compared the ΔR_g^{SAS} values with results from standard ff14SB or CHARMM36m (Fig. S16).[7] Upon modifying the LJ interaction with CUFIX in ff14SB simulations, (i) the density of Na^+ at the protein surface decreased relative to ff14SB and took lower densities as compared to ff99SB-ildn (Fig. S16g), (ii) ΔR_g^{SAS} values increased by ~0.2Å or ~0.1Å for SANS/D_2O or SANS/H_2O (Fig. S16b/c, pink vs. orange bars). However, the ΔR_g^{SAS} values with ff14SB/CUFIX remained below the results from CHARMM36m. Thus, refined Na^+–carboxylate Lennard-Jones interactions have only a small effect on ΔR_g^{SAS} values, and the increased ΔR_g^{SAS} value with CHARMM36m relative to several AMBER force fields is only partly explained by reduced binding of Na^+ ions to the protein surface.

Density correction for accurate buffer matching is critical for R_g predictions

Explicit-solvent SAS predictions implemented by GROMACS-SWAXS use a density correction for two reasons:[9,10] (i) Certain water models such as TIP3P underestimate the water density, which would lead to an overestimation of the contrast of the protein relative to bulk solvent and, thereby, to an overestimation of the forward scattering I_0. (ii) Since the bulk solvent density in the protein simulation may marginally differ from the density in the pure-solvent simulations, possibly owing to finite-size effects, spurious contrasts may emerge

at larger distances from the protein, which would lead to artifacts in the R_g estimate.

To avoid such artifacts, GROMACS-SWAXS corrects the solvent density both in the pure-solvent and in the protein simulation system. In the pure-solvent system, a uniform density is added to match a preselected bulk density ρ^0_{bulk}, which may be taken as the experimental density of pure water of $334\,\text{e}\,\text{nm}^{-3}$. In the protein simulation, the bulk density $\rho^{\text{MD}}_{\text{bulk}}$ is obtained by averaging over simulation frames within the volume outside of the envelope. A correction factor is obtained as the difference relative to ρ^0_{bulk} as $f = \rho^0_{\text{bulk}}/\rho^{\text{MD}}_{\text{bulk}}$ (see also Fig. S7a versus b). Then the solvent density inside of the envelope (which contributes to the calculated SAS curve) is corrected by the factor f.

Here, we tested the effect of the density correction on $\Delta R^{\text{SAS}}_{\text{g}}$ values. To this end, we first replaced the preselected bulk density of $334\,\text{e}\,\text{nm}^{-3}$ with the bulk density of the respective water model. Thereby, the protein–bulk contrast may not match the experimental value, however, accurate buffer matching between the protein and the pure-solvent simulations was still active. As expected, this led to a considerable change of the forward scattering I_0 for water models with inaccurate bulk densities such as TIP3P or cTIP3P; however, the $\Delta R^{\text{SAS}}_{\text{g}}$ revealed only a marginal change that was not visible in the bar plots.

Second, we fully disabled the density correction, thereby not ensuring accurate buffer matching any more. In these calculations, we observed changes of $\Delta R^{\text{SAS}}_{\text{g}}$ of SAXS relative to SANS/D_2O by up to 0.7 Å. In addition, the results revealed poor agreement with experiments. Thus, for accurate $\Delta R^{\text{SAS}}_{\text{g}}$ and for comparison with experiments, corrections for accurate buffer matching are mandatory, as used in the present study.

Supplementary Methods

Solvent density profiles around restrained bulk water

As described by Merzel and Smith,[11] solvent density profiles around proteins reflect not only effects from water–protein interactions, but furthermore effects owing to the internal structure of water. In a similar spirit to the analysis by Merzel and Smith, we compared

solvent density profiles around xylanase with density profiles around bulk water atoms taking the approximate shape of xylanase. To this end, we superimposed an envelope built at the Van-der-Waals surface of xylanase with bulk water simulations, and selected all water molecules within this envelope (GROMACS-SWAXS output file: excludedvolume_0.pdb). These water molecules were restrained in the following by restraining the oxygen atoms with harmonic potentials (force constant $1000\,\mathrm{kJ/mol/nm^2}$), henceforth referred to as restrained water (Fig. S8f, blue/white spheres). Next, simulations were set up as described in the Methods section in the main text, however, using the restrained water instead of the protein, and using the force field combinations c36–cTIP3P, ff15fb–TIP3P-FB, or ff99SBws–TIP4P/2005s. The geometries of restrained water molecules were constrained by using LINCS. Each system was simulated for 50 ns.

Density profiles (Fig. S8a) were computed from simulations of restrained water with the `gmx genenv` module of GROMACS-SWAXS, by using envelopes with 81920 triangular faces (option `-nrec 6`). The tool built a series of envelopes with increasing distances from the Van-der-Waals surface of the restrained water and computed the electron densities between pairs of adjacent envelopes.[12]

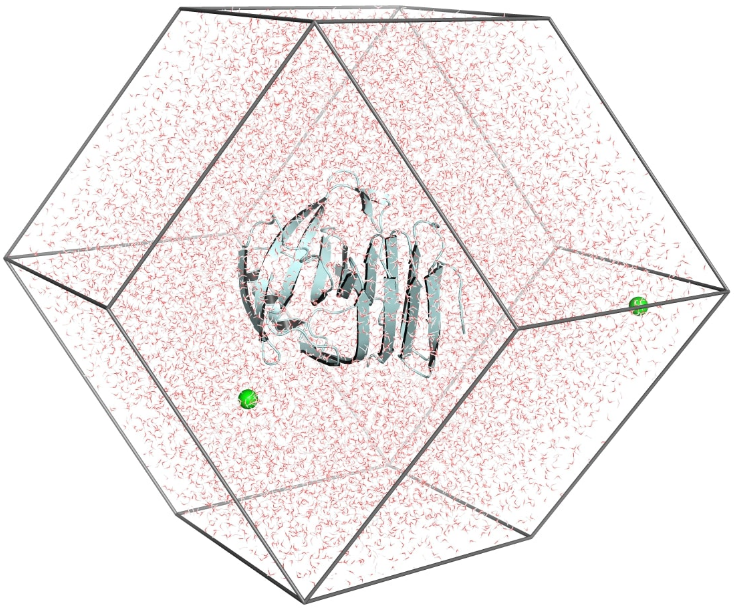

Figure S1: Simulation system of xylanase simulated with ff99SBws and TIP4P/2005s water in dodecahedral box. The protein is shown in cartoon representation, ions as spheres, and water as sticks. The box surfaces have a distance of at least 2 nm from the protein atoms.

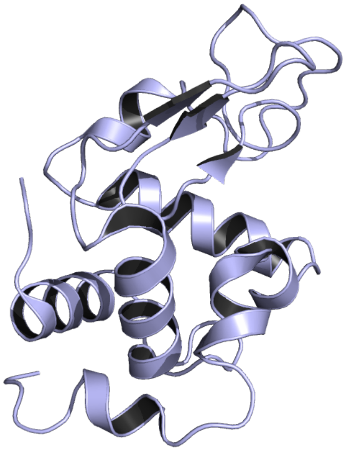

(a) Hen egg white lysozyme (PDB code 2VB1[13])

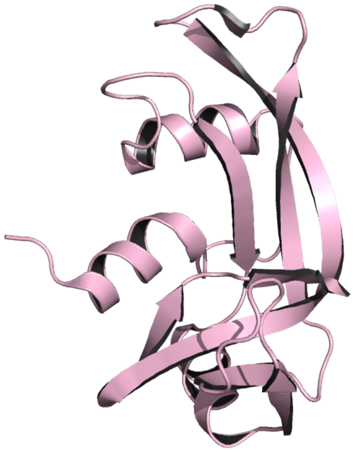

(b) Ribonuclease A (RNaseA; 7RSA[14])

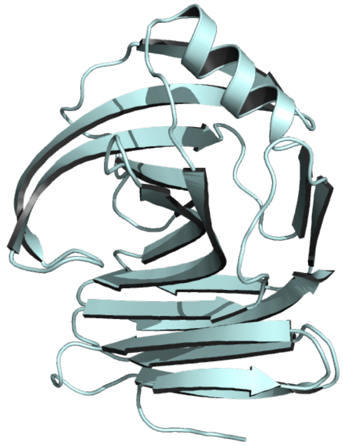

(c) Xylanase (2DFC[15])

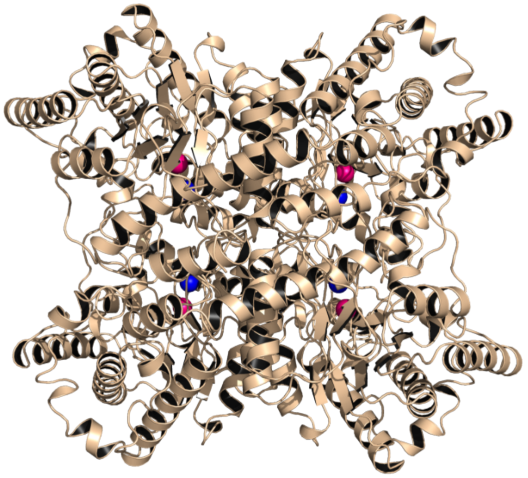

(d) Glucose isomerase. 1MNZ[16] after adding one methionine residue at the N- terminus. Ca^{2+} and Mg^{2+} ions inside the protein are shown as blue and pink spheres, respectively.

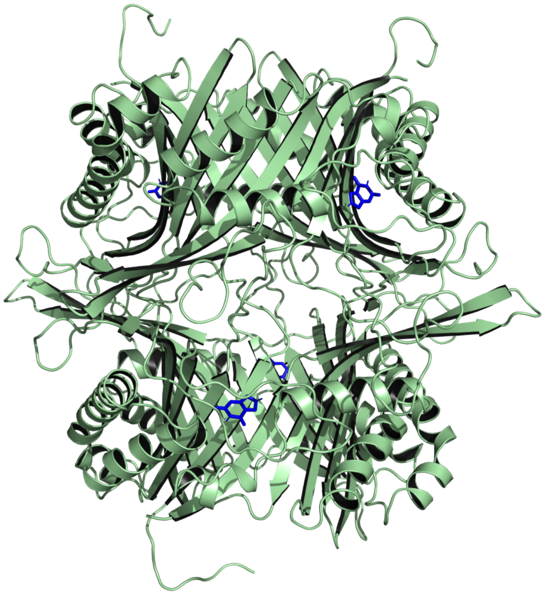

(e) Urate oxidase. 3L8W,[17] after adding six missing residues (sequence SLKSKL) at the C-terminus. Xanthine ligands are shown with blue sticks.

Figure S2: Cartoon representation of five proteins considered in this study (a) hen egg white lysozyme, (b) ribonuclease A (RNaseA), (c) xylanase , (d) glucose isomerase, and (e) urate oxidase.

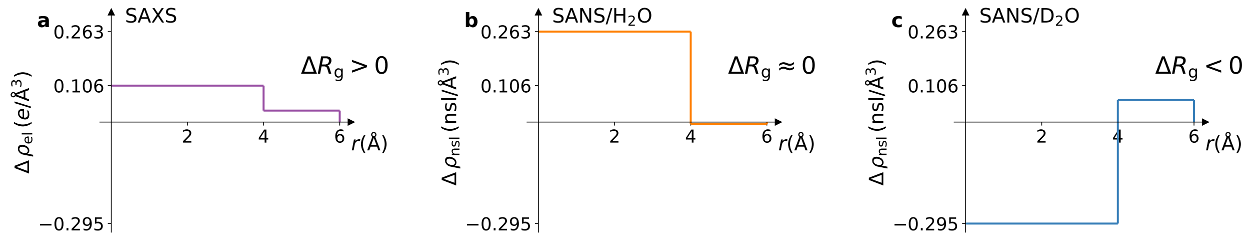

Figure S3: On the effect of the protein hydration shell on R_g as detected by SAXS, SANS in H_2O, or SANS in D_2O, illustrated with a hypothetical spherical protein with a (unitless) radius of 4 and a thickness of the hydration shell of 2. (a–c) Scattering contrast relative to bulk as function of distance r from the sphere center detected by (a) SAXS, (b) SANS/H_2O, and (c) SANS/D_2O experiments. (a) As detected by SAXS, both the protein and the hydration shell exhibit a positive electron density contrast relative to the bulk, resulting in an increased R_g owing to the hydration shell ($\Delta R_\mathrm{g} > 0$). (b) During SANS/H_2O, the protein exhibits a positive contrast of the neutron scattering length density, while the contrast of the hydration shell is close to zero, leading to a small influence by the hydration shell on R_g ($\Delta R_\mathrm{g} \approx 0$). (c) During SANS/$D_2O$, the protein exhibits a negative contrast whereas the hydration shell exhibits a positive contrast relative to bulk, resulting in a decreased R_g ($\Delta R_\mathrm{g} < 0$).

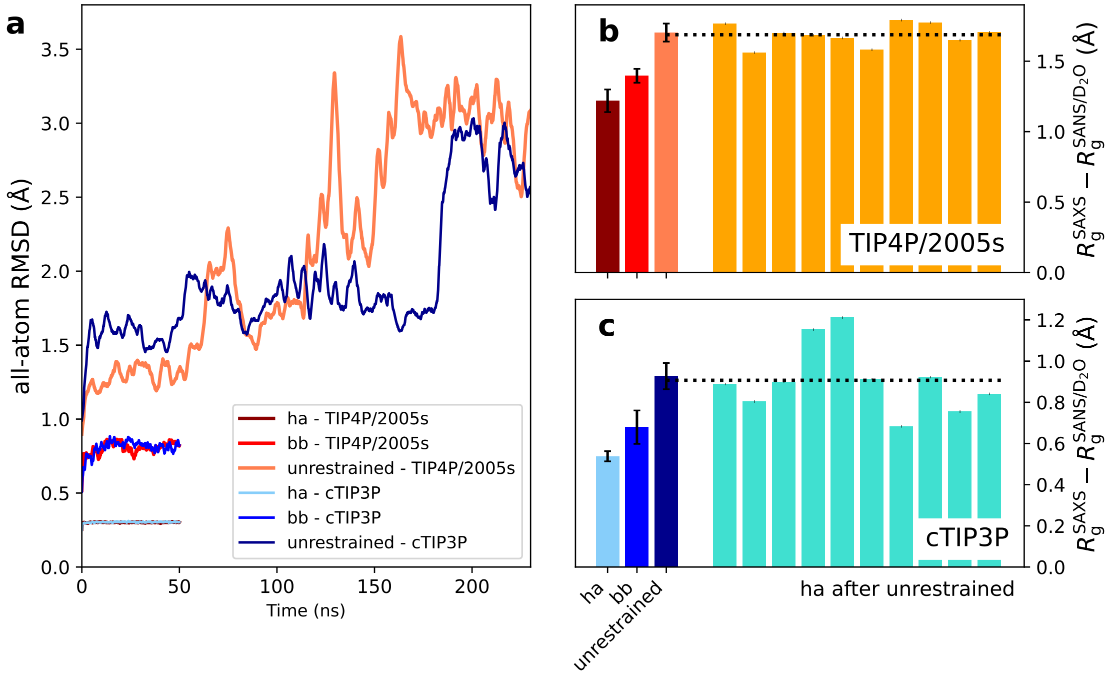

Figure S4: Effects of protein flexibility and hydration shell relaxation on R_g in simulations of xylanase. (a) All-atom RMSD with ff99SBws–TIP4P/2005s (brown shades) or CHARMM36m–cTIP3P (blue shades) with increasing flexibility. Simulations were carried out with restraints on heavy atoms (ha, see legend), with restraints on the backbone (bb), or without restraints (unrestrained). (b) Difference $\Delta R_\mathrm{g}^\mathrm{SAS}$ between R_g values from SAXS and SANS/D_2O for amber99SBws–TIP4P/2005s and (c) CHARMM36m–cTIP3P. Left three bars: $\Delta R_\mathrm{g}^\mathrm{SAS}$ from simulations with restraints on heavy atoms, restraints on the backbone, or from unrestrained simulations starting from the crystal structure (color code according to panel a), revealing an increased $\Delta R_\mathrm{g}^\mathrm{SAS}$ with increasing protein flexibility. Right ten bars: $\Delta R_\mathrm{g}^\mathrm{SAS}$ values from MD simulations with restraints on all heavy atoms starting from different times of the unrestrained simulation. Critically, the average $\Delta R_\mathrm{g}^\mathrm{SAS}$ values from these ten restrained simulations (dotted lines) agree with $\Delta R_\mathrm{g}^\mathrm{SAS}$ from the initial unrestrained simulation. Thus, not the protein flexibility per se, but instead the relaxation of protein–water packing during the initial unrestrained MD leads on average to increased $\Delta R_\mathrm{g}^\mathrm{SAS}$ values.

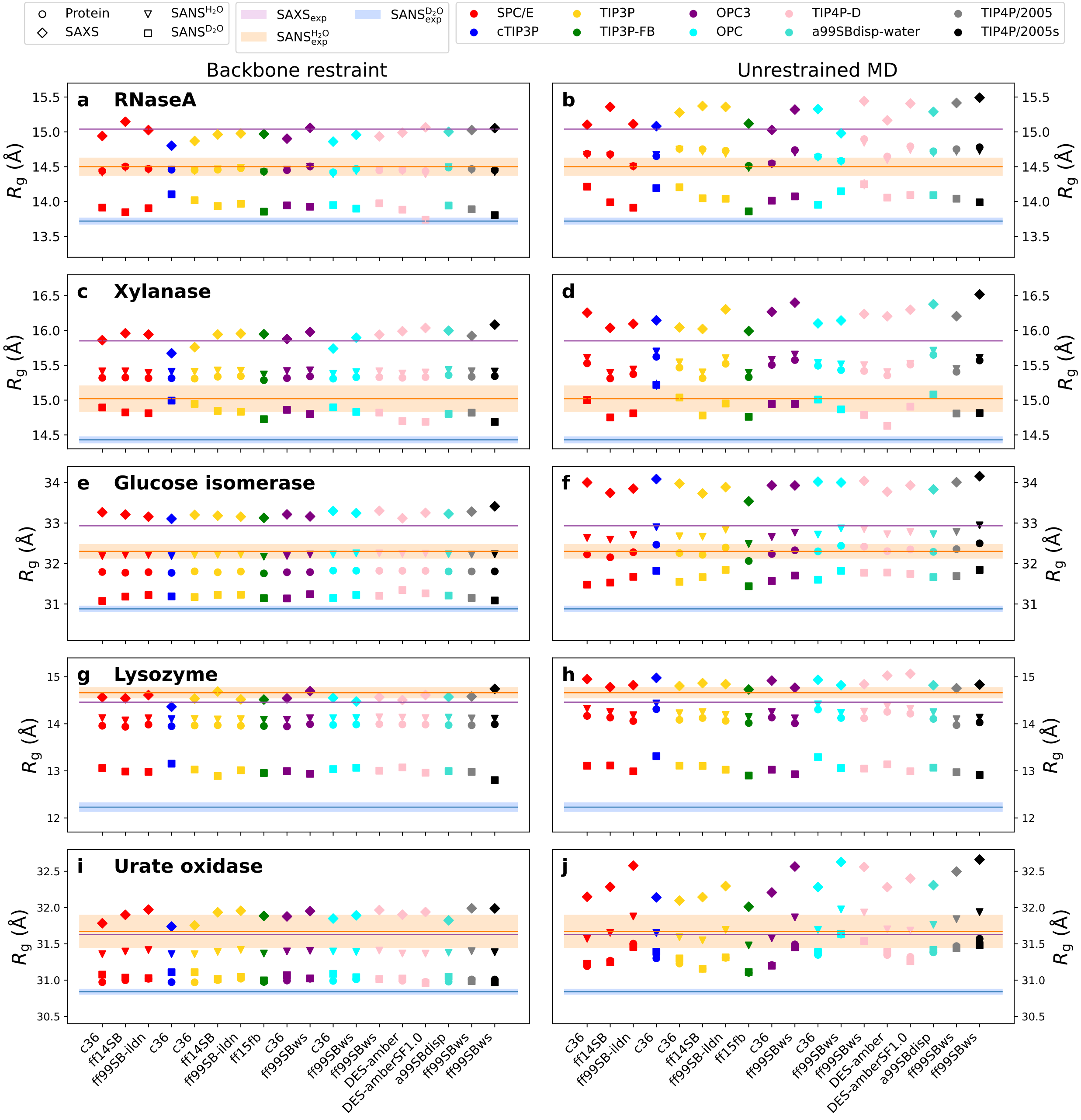

Figure S5: Absolute R_g values of (a/b) RNaseA, (c/d) xylanase, (e/f) glucose isomerase, (g/h) lysozyme, and (i/j) urate oxidase obtained from MD simulations with backbone restraints (left column) and without restraints (right column) with 18 different combinations of protein force fields (labels along the abscissa) and water models (color code, see legend). R_g^{SAS} values from unrestrained MD simulations for SAXS (diamonds), SANS/H_2O (triangles), and SANS/D_2O (squares). R_g of the pure protein (R_g^{Prot}) shown as circles. Experimental R_g^{SAS} values from $P(r)$ analysis are shown has horizontal lines for SAXS (purple), SANS/H_2O (orange), and SANS/D_2O blue. Shaded areas indicated experimental uncertainties of consensus SAS data.[1] Absolute R_g values shown here are not suitable for validating the hydration shell against experimental data because R_g of the bare protein (R_g^{Prot}) is not accurately known in the experiment.

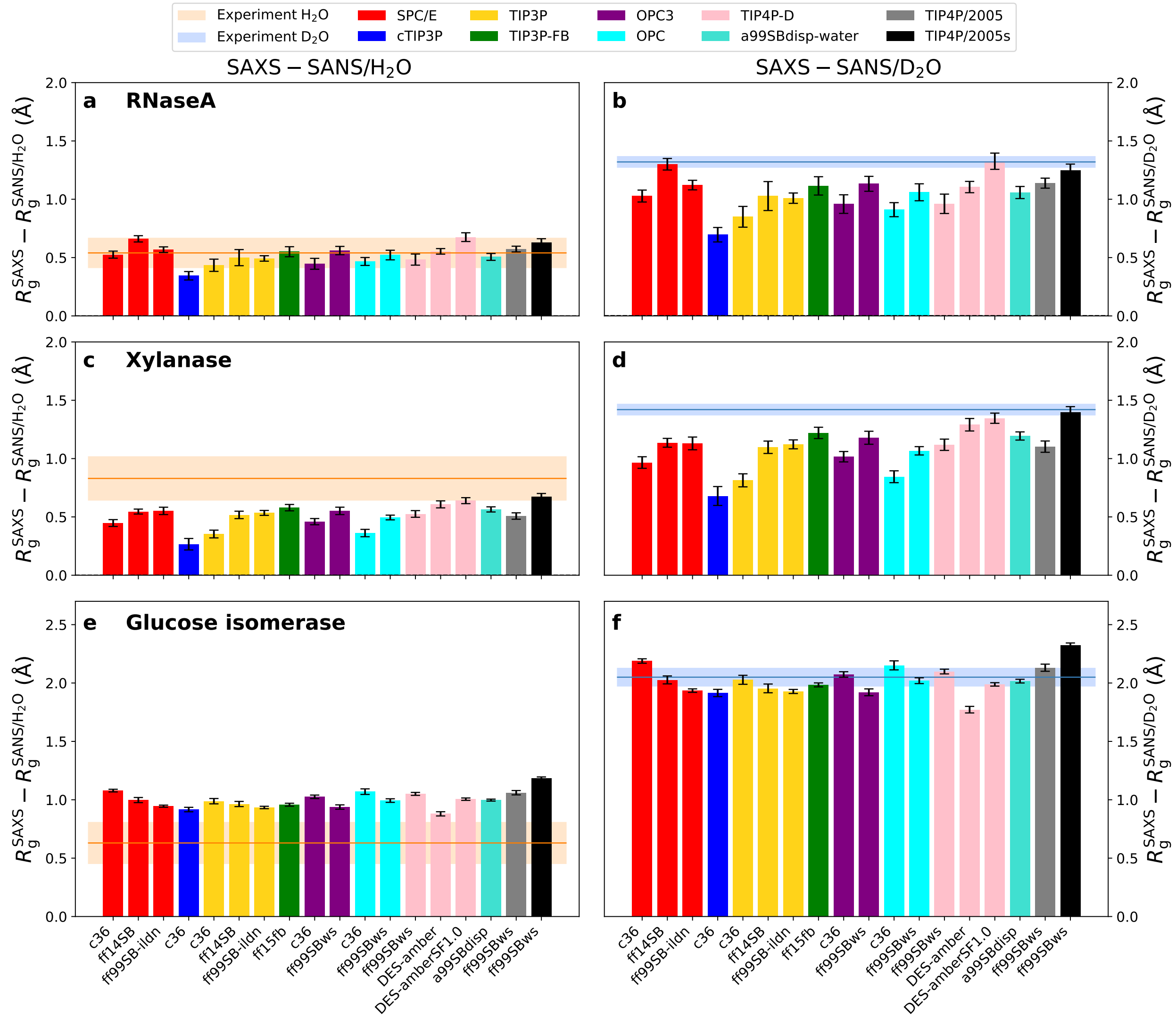

Figure S6: Same analysis as shown in Fig. 3, however based on simulations with backbone restraints instead of based on unrestrained simulations. Large variations of ΔR_g^{SAS} among different force field combinations are evident, even with identical backbone conformations. Thus, differences in protein–water interactions among different force fields and not different protein conformations in unrestrained simulations dominate the variations of ΔR_g^{SAS}. As a second finding, ΔR_g^{SAS} values from backbone-restrained simulations (this figure) are mostly smaller as compared to values from unrestrained simulations (Fig. 3), demonstrating that the equilibration of protein–water interactions during unrestrained simulations leads to a (slightly) increased packing of the hydration shell (see also Fig. S4).

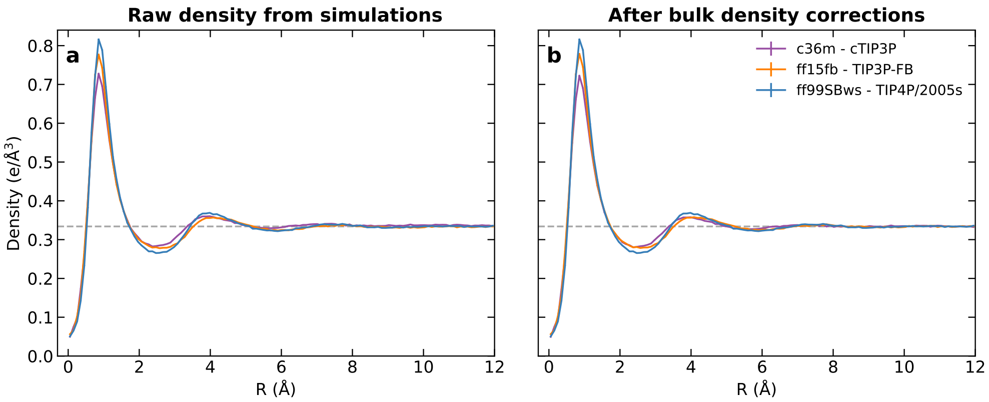

Figure S7: Typical solvent densities versus distance R from the Van-der-Waals surface and averaged over the protein surface, here shown for the protein xylanase obtained from simulations with c36–cTIP3P (purple), ff15fb–TIP3P-FB (orange), or ff99SBws–TIP4P/2005s (blue). (a) Densities taken from MD simulations or (b) densities scaled by a constant factor to correct the bulk density to $334\,\mathrm{e/nm^{-3}}$ (dashed horizontal line). The peaks representing the three hydration layers differ among the three combinations of protein force field and water model. The combination ff99SBws–TIP4P/2005s leads to the most pronounced hydration layer peaks, while c36–cTIP3P to the least pronounced peaks, which correlates with the effect of the hydration shells on R_g (compare with ΔR_g values in Fig. 2). Densities were computed with the gmx genenv module of GROMACS-SWAXS.

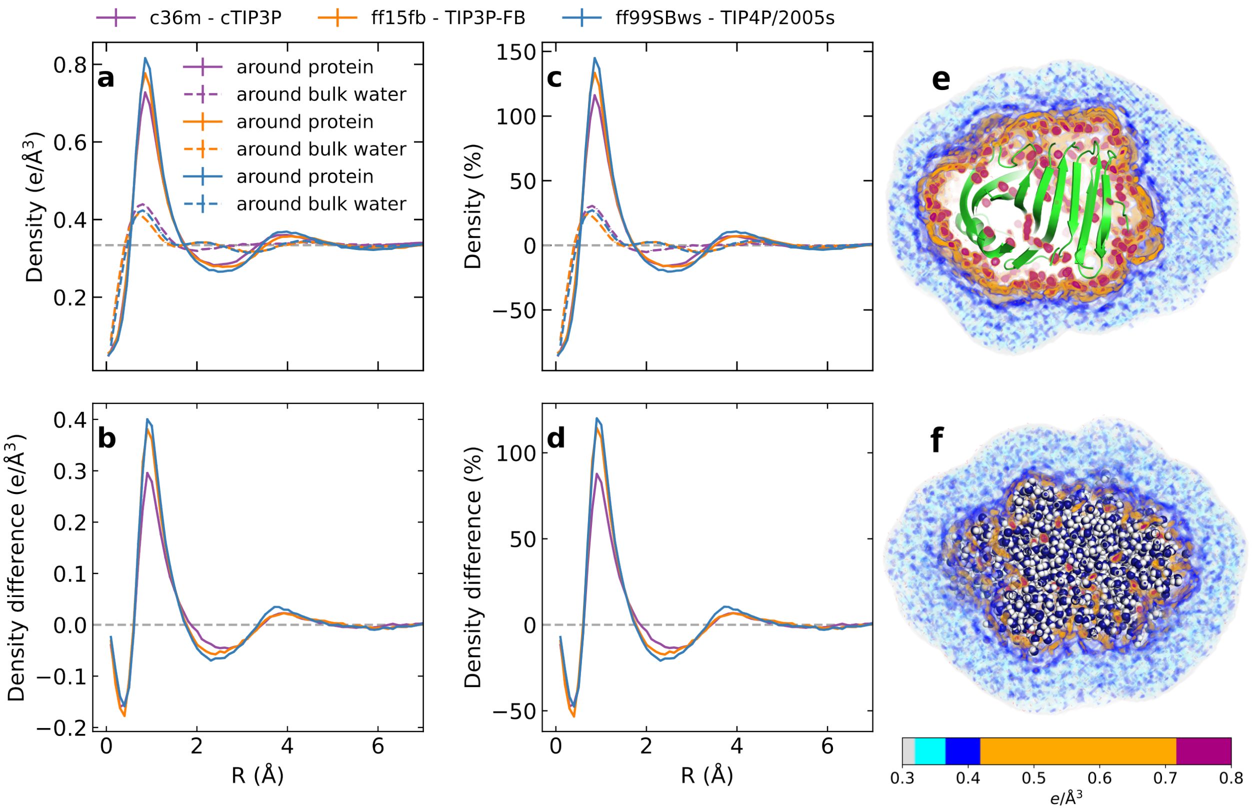

Figure S8: Solvent density modulations owing to water–protein interactions are by far larger as compared to density modulations owing to the internal structure of bulk water: (a) Solid lines: solvent density versus distance R from the Van-der-Waals surface of xylanase and averaged over the protein surface for c36–cTIP3P (purple), ff15fb–TIP3P-FB (orange), or ff99SBws–TIP4P/2005s (blue). Dashed colored lines: solvent density versus distance R from a volume of restrained bulk water atoms, taking approximately the shape of xylanase (see panels e and f). Dashed grey line: experimental bulk solvent density of $0.334\,e/\text{Å}^3$. (b) Density difference profiles of density around xylanase relative to density around bulk water, computed as the difference between the solid and dashed lines of panel a. The marked density difference manifests in an increased R_g as detected by SAXS. (c/d) Same analyses as in panels a/b, however, plotted as the relative difference of the density, similar to the analysis presented by Merzel and Smith.[11] (e) Electron density of solvent around xylanse with restrained heavy atoms (green cartoon) and (f) around bulk water with restrained oxygen atoms (blue/white spheres). Solvent densities are visualized in shades from light grey (bulk water) to blue to orange (see color bar), highlighting a by far more pronounced hydration shell around xylanase as compared to the solvent around bulk water. Simulations of the restrained solvent molecules inside the protein volume were performed as described in the Supplementary Methods. Densities were computed with the gmx genenv module of GROMACS-SWAXS.

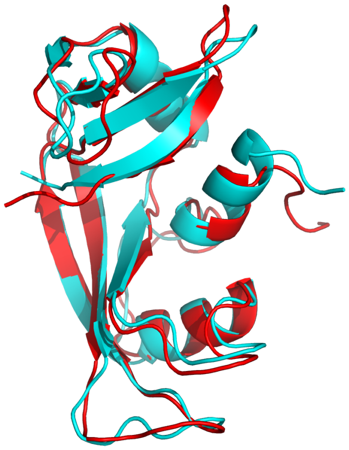

Figure S9: Structures of RNaseA in simulations with CHARMM36m–OPC after equilibration (turquoise) and at ∼100 ns of unrestrained MD simulation (red). The N-terminal helix partly unfolded in the unrestrained simulation. In simulation with other force field combinations, the N-terminal helix exhibited some flexibility as well, however, the helix re-folded during the simulations. The conformational instability of RNaseA in CHARMM36m–OPC simulations may explain the the large $\Delta R_{\mathrm{g}}^{\mathrm{SAS}}$ value in unrestrained simulations of RNAseA (Fig. 3b, light turquoise bar labeled with c36), in contrast to all other unrestrained simulations with CHARMM36m (see Fig. 3) or all restrained simulations CHARMM36m (including CHARMM36m–OPC, see Fig. S6).

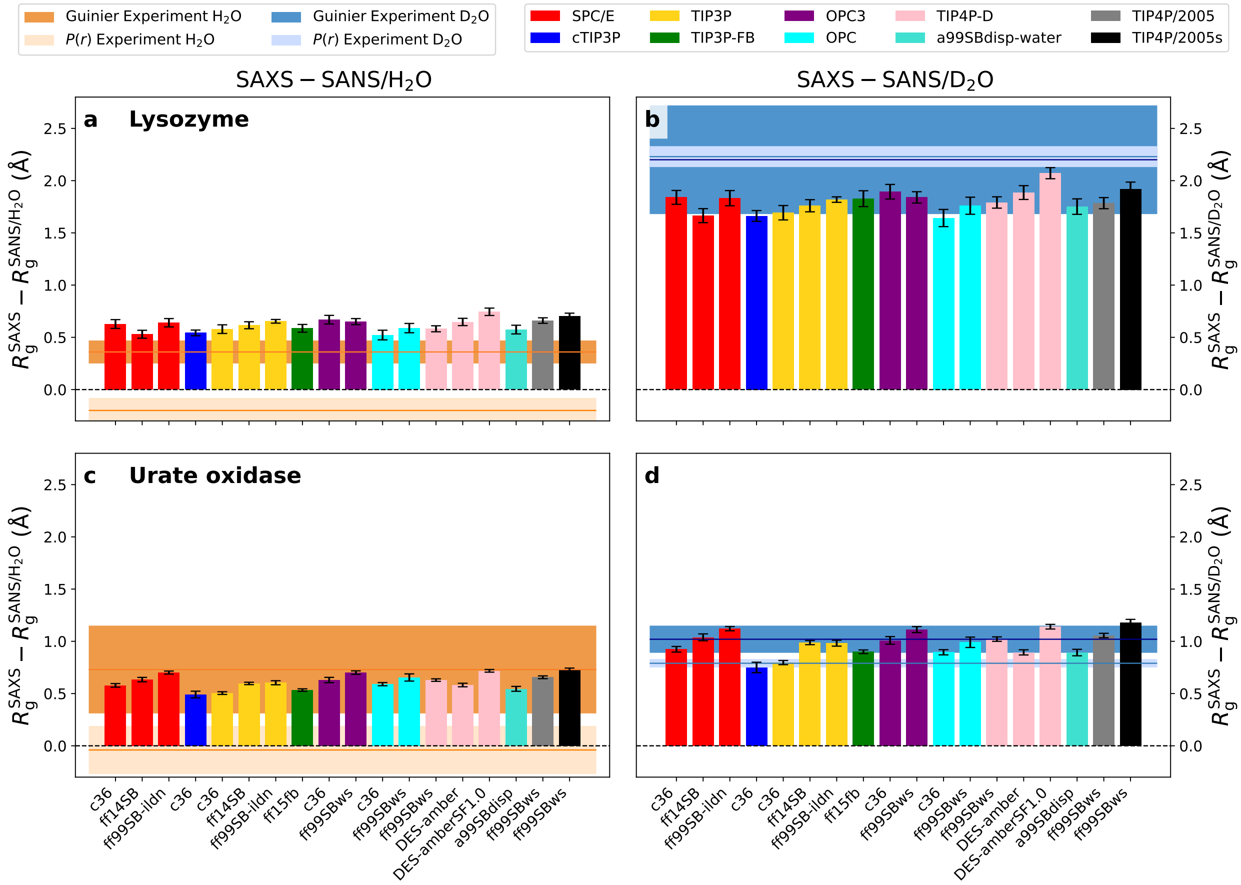

Figure S10: ΔR_g^{SAS} values of (a/b) lysozyme and (c/d) urate oxidase for SAXS relative to SANS/H_2O (left column) and SAXS relative to SANS/D_2O (right column). Experimental consensus data (horizontal lines) and reported uncertainties (shaded areas) from Guinier analysis (dark brown, dark blue) and from $P(r)$ analysis (light brown, light blue) are shown for reference. Experimental values for lysozyme and urate oxidase are subject to increased uncertainty, owing to problems with radiation damage and aggregation (see Supplementary Discussion).[1]

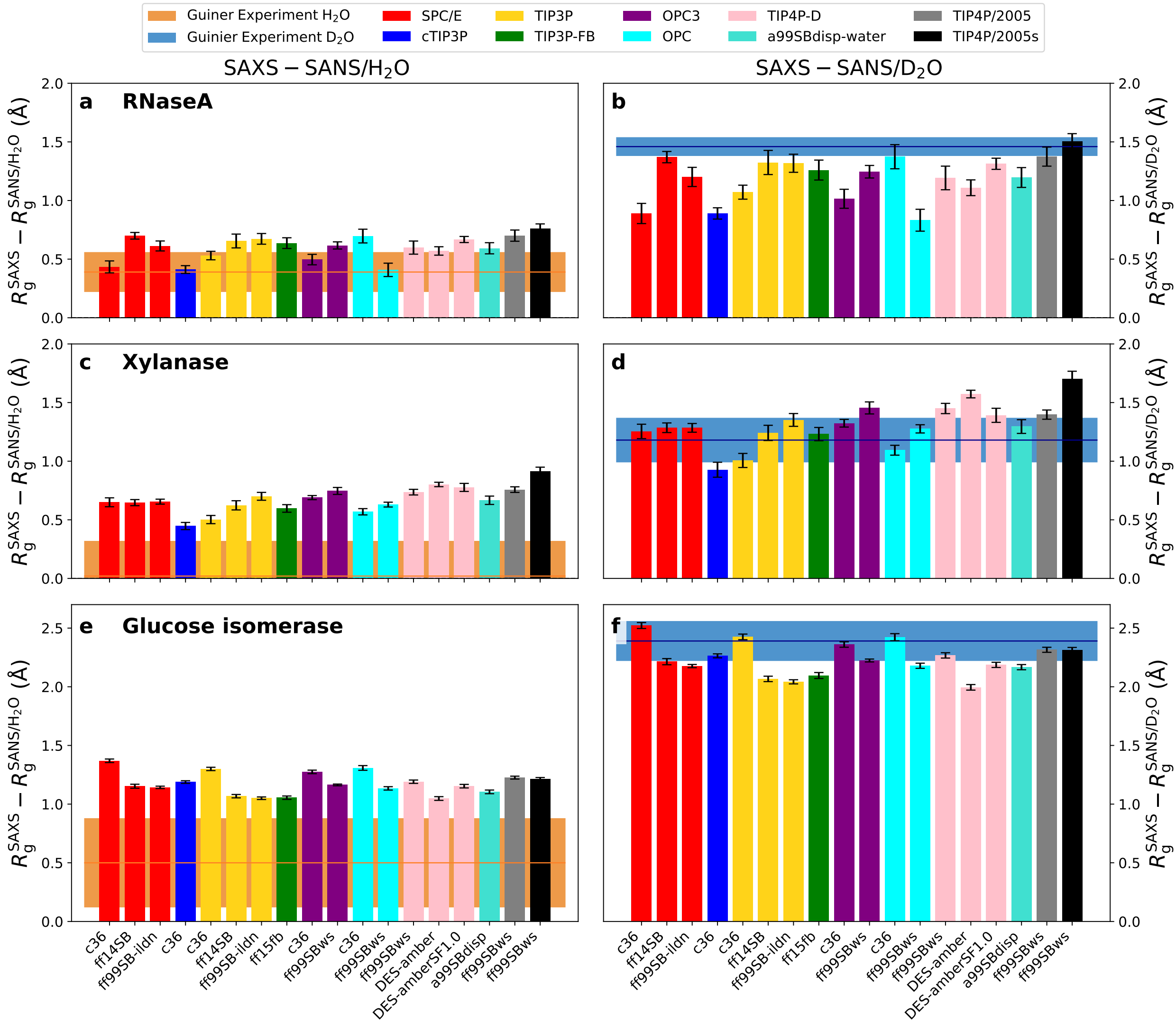

Figure S11: ΔR_g^{SAS} for (a/b) RNaseA, (c/d) xylanase, and (e/f) glucose isomerase, following the same labeling and color code as in Fig. 3. Experimental values obtained form Guinier analysis are shown as horizontal lines with uncertainties as shaded areas,[1] in contrast to experimental values from the $P(r)$ function in Fig. 3. The larger experimental errors as compared to the results from $P(r)$ analysis and the slightly poorer agreement between simulations and experiment (compared to Fig. 3) are rationalized by the fact that Guinier analysis is more sensitive to small amounts of undetected protein–protein aggregation.[1] Left column: ΔR_g^{SAS} from SAXS relative to SANS/H_2O revealing poor agreement between simulation and experiment, likely caused by poorer signal-to-noise ratio during SANS/H_2O experiments owing to increased incoherent scattering in H_2O as compared to D_2O and lower contrast.

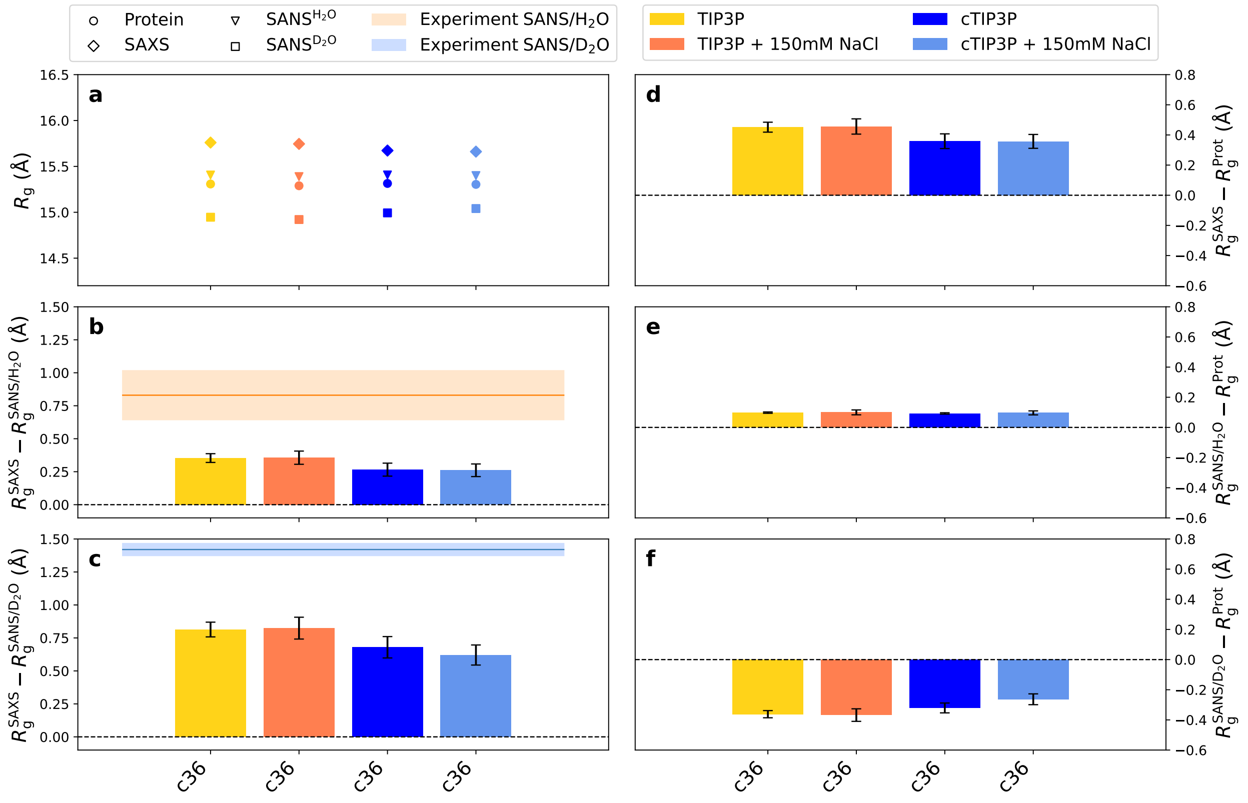

Figure S12: On the effect of 150 mM NaCl on SAS-derived R_g values of xylanase as compared to simulations with purely counter ions. Simulations were carried out with Charmm36m combined with three different water models using purely counter ions (SPC/E, TIP3P, or cTIP3P, see legend for color code) or with 150 mM NaCl (denoted SPC/E-ions, TIP3P-ions, cTIP3P-ions, see legend). (a) SAS-derived R_g^{SAS} computed from unrestrained MD simulations for SAXS (diamonds), SANS/H_2O (triangles), and SANS/D_2O (squares). R_g of the pure protein (R_g^{Prot}) shown as circles. Experimental R_g^{SAS} values from $P(r)$ analysis are shown has horizontal lines for SAXS (purple), SANS/H_2O (orange), and SANS/D_2O (blue). (b) Difference between R_g values from SAXS and SANS/H_2O as well as (c) between SAXS and SANS/D_2O. (d) Difference between SAS-derived R_g values from explicit-solvent MD and R_g^{Prot} values for SAXS,(e) for SANS/H_2O, and (f) for SANS/D_2O. Statistical errors (1 SE) were obtained from block averaging. Horizontal lines and shaded areas indicated experimental consensus values and uncertainties.[1] Adding 150 mM NaCl to the buffer has only a small effect on the ΔR_g values of the only slightly charged xylanase.

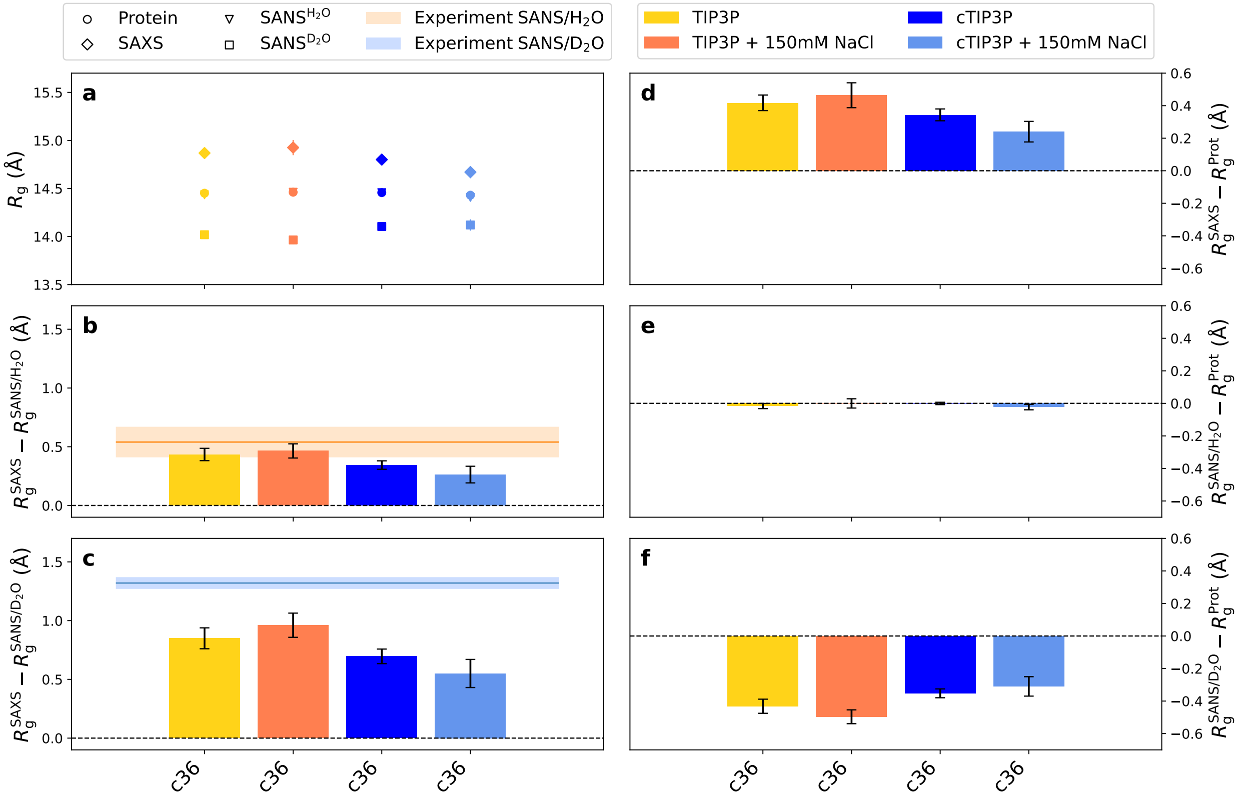

Figure S13: On the effect of 150 mM NaCl on SAS-derived R_g values of RNaseA. Same analysis and as shown for xylanase in Fig. S12.

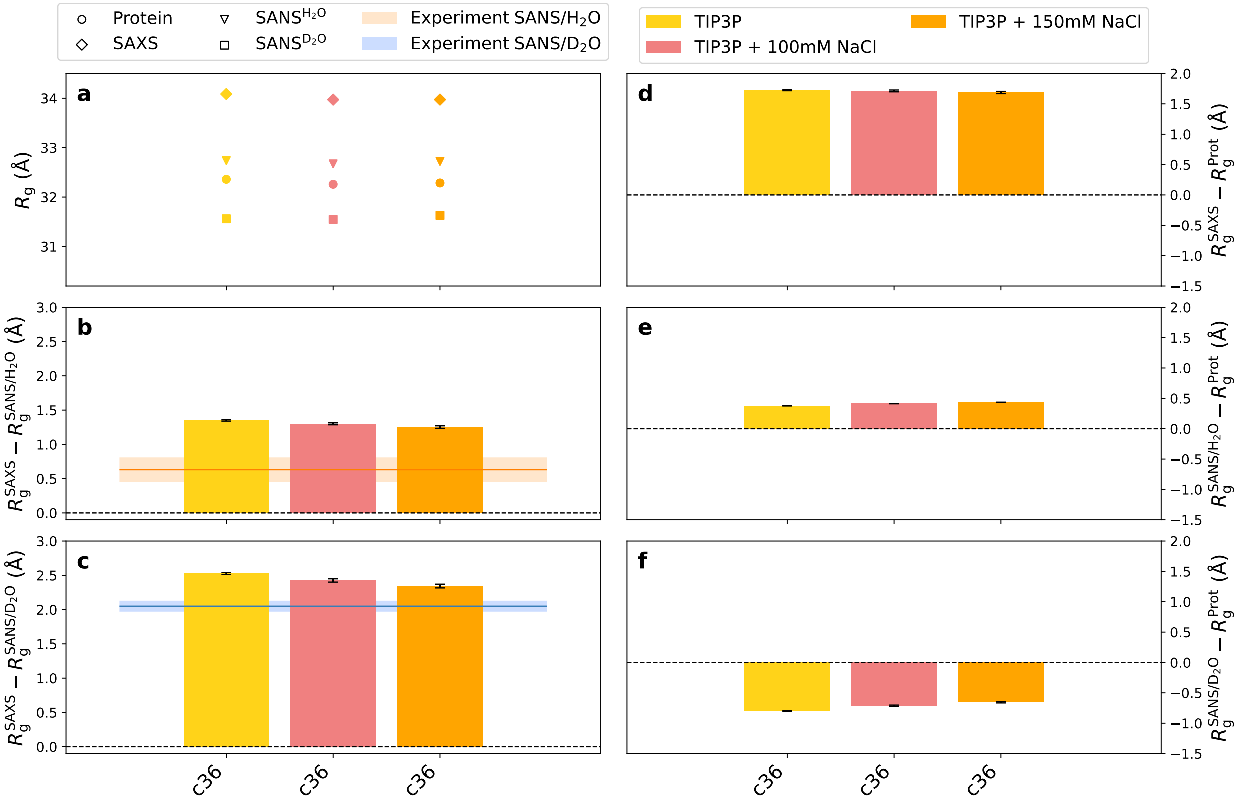

Figure S14: On the effect of 100 mM NaCl (pink) or 150 mM NaCl (orange) on SAS-derived R_g values of the highly anionic glucose isomerase ($-60\,e$), as compared to using only counter ions (yellow). Same analysis as shown for xylanase and RNaseA in Figs. S12 and S13, however restricted to TIP3P water.The lower the ion concentration in the buffer, the larger the Debye length and thus the effect of the hydration shell on the R_g.

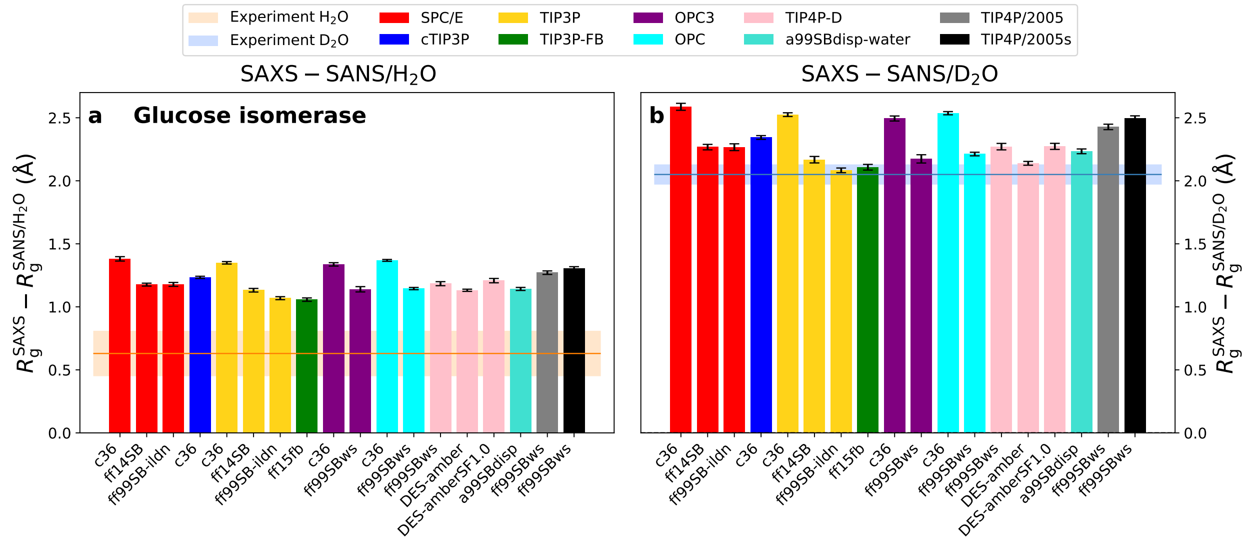

Figure S15: On the importance of salt for accurate R_g predictions of highly anionic glucose isomerase. (a) $\Delta R_\mathrm{g}^\mathrm{SAS}$ values from SAXS relative to SANS/H_2O and (b) of SAXS relative to SANS/D_2O from unrestrained simulations of glucose isomerase with only counter ions (60 Na^+) but no additional salt. Experimental consensus values and uncertainties from $P(r)$ analysis are shown as horizontal lines and shaded areas, respectively. By comparison with Fig. 3e/f, the lack of salt increases $\Delta R_\mathrm{g}^\mathrm{SAS}$ systematically, caused by an overly extended counter ion cloud. In turn, in presence of salt, the Debye length decreases, thus leading to a spatially more compact counter ion cloud and slightly smaller $\Delta R_\mathrm{g}^\mathrm{SAS}$ values (compare with Fig. 3).

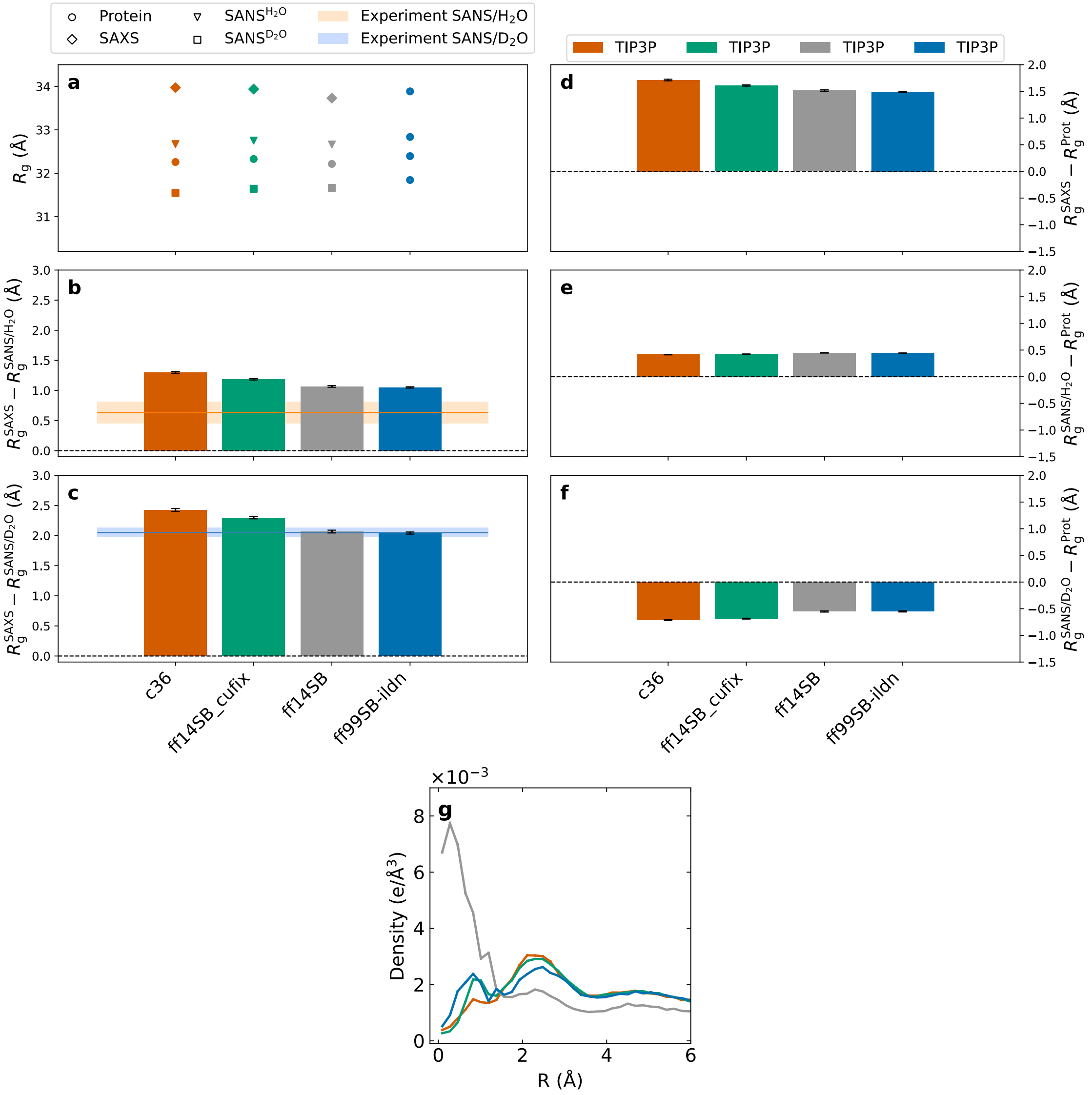

Figure S16: On the effect of non-bonded fix parameters (CUFIX, also called NBFIX) with the Amber14SB protein force field. CUFIX implements modified Lennard-Jones interactions between Na^+ ions and carboxylate oxygen atoms of aspartate and glutamate residues with the aim to avoid Na^+–COO^- overbinding.[7] CHARMM36m implements CUFIX by default in the version of July 2020 as used here. (a) R_g, (b/c) ΔR_g^{SAS}, and (d–f) ΔR_g values are reported for the highly anionic glucose isomerase (GI, $-60\,e$). Unrestrained simulations of GI were carried out with CHARMM36m (yellow bar), ff14SB with CUFIX (pink), as well as ff14SB or ff99SB-ildn without CUFIX (grey and blue, respectively). Experimental consensus values and uncertainties from $P(r)$ analysis are shown as horizontal lines and shaded areas, respectively. Statistical errors (1 SE) were obtained from block averaging. Upon refining Na^+–carboxylate interactions by ff14SB with CUFIX, ΔR_g^{SAS} increased by only ~0.2 Å and ~0.1Å for SANS in D_2O and H_2O, respectively. (g) Density of Na^+ as function of distance from the protein surface for four different force fields (color code according to panels a–f). CUFIX greatly reduces Na^+ overbinding in ff14SB simulations.

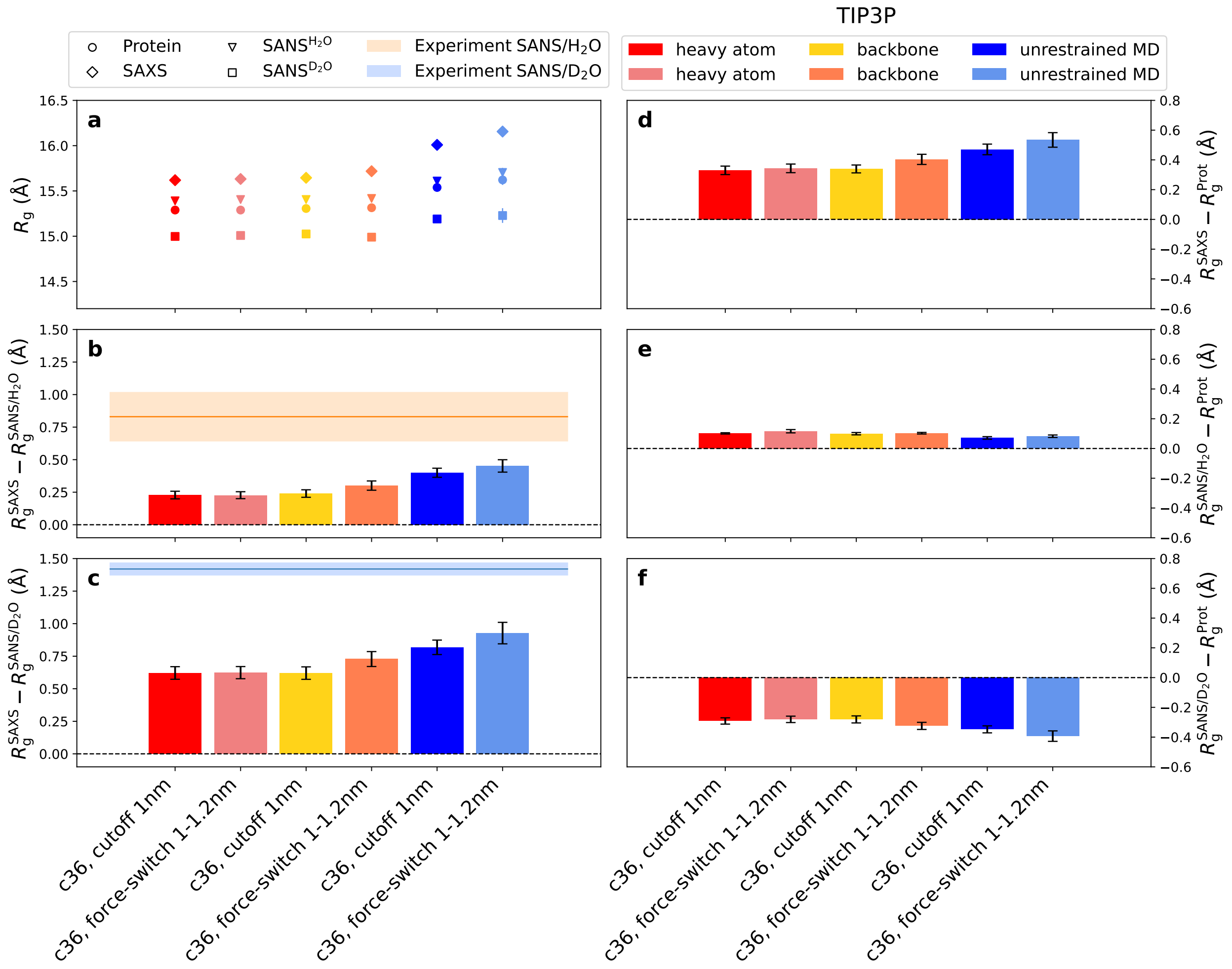

Figure S17: On the role of force field-specific Lennard-Jones (LJ) cutoff settings: (a) R_g, (b/c) ΔR_g^{SAS}, and (d–f) ΔR_g values for xylanase using the CHARMM36m–TIP3P force field combination. Simulations were carried out either using a plain LJ cutoff at 1.0 nm or by switching off the LJ forces between 1 nm and 1.2 nm, as recommended for the CHARMM36m force field (force-switch 1–1.2 nm). Results are shown for simulations with restrained heavy atoms (red, pink), restrained backbone (yellow, orange) or for unrestrained MD (dark and light blue). In simulations with flexible side chains (unrestrained or backbone-restrained MD), cutoff settings following CHARMM standards leads to larger ΔR_g^{SAS} values as compared to using a plain 1 nm cutoff (panels b/c, orange vs. yellow, light blue vs. dark blue). In simulations with restrained heavy atoms, using a different cutoff stetting has only a marginal effect (pink vs. red). Thus, the additional LJ interactions between 1 nm and 1.2 nm lead to increased ΔR_g^{SAS} values owing to more densely packed water structures around flexible protein side chains, and not owing to stronger attraction of water onto a (fixed) protein surface.

Table S1: Eighteen combinations of protein force field and water model used in this study. CHARMM36m was taken from version of July 2020.

Protein force field	Abbreviation	Water model	Refs.
CHARMM36m	c36	cTIP3P	18, 19
		TIP3P	20
		SPC/E	21
		OPC3	22
		OPC	23
Amber14SB	ff14SB	SPC/E	24 , 21
		TIP3P	20
Amber99SB-ildn	ff99SB-ildn	SPC/E	25, 21
		TIP3P	20
Amber15/force-balance	ff15fb	TIP3P-FB	26
Amber99SBws	ff99SBws	TIP4P/2005s	27, 28
		TIP4P/2005	29
		TIP4P-D	30
		OPC3	22
		OPC	23
DES-amber	DES-amber	TIP4P-D	31, 30
DES-amber without scaled charges	DES-amberSF1.0	TIP4P-D	31, 30
Amberr99SBdisp	a99SBdisp	a99SBdisp-water	32

Table S2: Experimental consensus R_g values for SAXS, SANS/H_2O, and SANS/D_2O from Trewhella et al.[1] R_g are shown as obtained from Guinier or $P(r)$ analysis. Two right columns: $\Delta R_\mathrm{g}^\mathrm{SAS}$ values from SAXS relative to SANS/H_2O and SAXS relative to SANS/D_2O, respectively.

Protein	Type of R_g	$R_\mathrm{g}^\mathrm{SAXS}$ (Å)	$R_\mathrm{g}^\mathrm{SANS/H_2O}$ (Å)	$R_\mathrm{g}^\mathrm{SANS/D_2O}$ (Å)	$R_\mathrm{g}^\mathrm{SAXS} - R_\mathrm{g}^\mathrm{SANS/H_2O}$ (Å)	$R_\mathrm{g}^\mathrm{SAXS} - R_\mathrm{g}^\mathrm{SANS/D_2O}$ (Å)
RNaseA	Guinier	15.13 ± 0.02	14.74 ± 0.17	13.67 ± 0.08	0.39 ± 0.17	1.46 ± 0.08
	$P(r)$	15.04 ± 0.01	14.50 ± 0.13	13.72 ± 0.05	0.54 ± 0.13	1.32 ± 0.05
Xylanase	Guinier	16.05 ± 0.01	16.03 ± 0.30	14.87 ± 0.19	0.02 ± 0.3	1.18 ± 0.19
	$P(r)$	15.85 ± 0.01	15.02 ± 0.19	14.43 ± 0.05	0.83 ± 0.19	1.42 ± 0.05
Glucose isomerase	Guinier	33.11 ± 0.05	32.61 ± 0.38	30.72 ± 0.17	0.5 ± 0.38	2.39 ± 0.17
	$P(r)$	32.93 ± 0.01	32.30 ± 0.18	30.88 ± 0.08	0.63 ± 0.18	2.05 ± 0.08
Lysozyme	Guinier	14.64 ± 0.05	14.28 ± 0.12	12.44 ± 0.52	0.36 ± 0.11	2.2 ± 0.52
	$P(r)$	14.46 ± 0.01	14.66 ± 0.12	12.23 ± 0.10	-0.2 ± 0.12	2.23 ± 0.1
Urate Oxidase	Guinier	32.30 ± 0.06	31.57 ± 0.42	31.18 ± 0.12	0.73 ± 0.42	1.02 ± 0.13
	$P(r)$	31.63 ± 0.01	31.67 ± 0.23	30.84 ± 0.04	-0.4 ± 0.23	0.79 ± 0.04

force field	water model	R_g^{Prot} (Å)	R_g^{SAXS} (Å)	R_g^{SANS/H_2O} (Å)	R_g^{SANS/D_2O} (Å)	$R_g^{SAXS} - R_g^{Prot}$ (Å)	$R_g^{SANS/H_2O} - R_g^{Prot}$ (Å)	$R_g^{SANS/D_2O} - R_g^{Prot}$ (Å)	$R_g^{SAXS} - R_g^{SANS/H_2O}$ (Å)	$R_g^{SAXS} - R_g^{SANS/D_2O}$ (Å)
c36	SPC/E	14.69 ± 0.03	15.10 ± 0.07	14.67 ± 0.03	14.21 ± 0.04	0.42 ± 0.06	-0.02 ± 0.02	-0.47 ± 0.04	0.43 ± 0.06	0.89 ± 0.09
ff14SB	SPC/E	14.68 ± 0.03	15.36 ± 0.04	14.66 ± 0.03	13.99 ± 0.04	0.68 ± 0.03	-0.02 ± 0.01	-0.69 ± 0.03	0.70 ± 0.03	1.37 ± 0.05
ff99SB-ildn	SPC/E	14.51 ± 0.02	15.11 ± 0.06	14.50 ± 0.03	13.91 ± 0.05	0.60 ± 0.05	-0.01 ± 0.02	-0.60 ± 0.04	0.61 ± 0.05	1.20 ± 0.09
c36	cTIP3P	14.65 ± 0.05	15.08 ± 0.06	14.67 ± 0.05	14.19 ± 0.04	0.43 ± 0.04	0.02 ± 0.02	-0.46 ± 0.03	0.41 ± 0.04	0.89 ± 0.05
c36	TIP3P	14.76 ± 0.03	15.28 ± 0.05	14.75 ± 0.03	14.21 ± 0.04	0.52 ± 0.04	-0.01 ± 0.02	-0.55 ± 0.04	0.53 ± 0.04	1.07 ± 0.06
ff14SB	TIP3P	14.75 ± 0.02	15.37 ± 0.07	14.72 ± 0.02	14.05 ± 0.05	0.62 ± 0.06	-0.03 ± 0.01	-0.70 ± 0.05	0.65 ± 0.06	1.32 ± 0.11
ff99SB-ildn	TIP3P	14.73 ± 0.02	15.36 ± 0.05	14.69 ± 0.02	14.04 ± 0.04	0.63 ± 0.05	-0.04 ± 0.02	-0.69 ± 0.04	0.67 ± 0.05	1.32 ± 0.08
ff15fb	TIP3P-FB	14.51 ± 0.02	15.12 ± 0.06	14.48 ± 0.02	13.86 ± 0.04	0.61 ± 0.05	-0.03 ± 0.02	-0.65 ± 0.05	0.64 ± 0.05	1.26 ± 0.09
c36	OPC3	14.55 ± 0.01	15.03 ± 0.05	14.53 ± 0.02	14.01 ± 0.05	0.48 ± 0.05	-0.02 ± 0.02	-0.53 ± 0.04	0.50 ± 0.05	1.01 ± 0.09
ff99SBws	OPC3	14.74 ± 0.03	15.32 ± 0.04	14.70 ± 0.03	14.07 ± 0.03	0.58 ± 0.04	-0.03 ± 0.02	-0.66 ± 0.03	0.62 ± 0.04	1.25 ± 0.06
c36	OPC	14.65 ± 0.02	15.33 ± 0.07	14.63 ± 0.02	13.95 ± 0.05	0.68 ± 0.06	-0.02 ± 0.02	-0.69 ± 0.06	0.70 ± 0.06	1.37 ± 0.11
ff99SBws	OPC	14.58 ± 0.04	14.98 ± 0.08	14.57 ± 0.03	14.15 ± 0.04	0.40 ± 0.06	-0.01 ± 0.01	-0.44 ± 0.04	0.41 ± 0.06	0.83 ± 0.10
ff99SBws	TIP4P-D	14.90 ± 0.06	15.44 ± 0.08	14.84 ± 0.06	14.25 ± 0.08	0.55 ± 0.06	-0.05 ± 0.02	-0.65 ± 0.05	0.60 ± 0.06	1.19 ± 0.11
DES-amber	TIP4P-D	14.65 ± 0.02	15.17 ± 0.05	14.60 ± 0.02	14.06 ± 0.03	0.52 ± 0.05	-0.05 ± 0.02	-0.59 ± 0.03	0.57 ± 0.04	1.11 ± 0.07
DES-amberSF1.0	TIP4P-D	14.79 ± 0.03	15.41 ± 0.04	14.74 ± 0.02	14.09 ± 0.03	0.61 ± 0.04	-0.05 ± 0.01	-0.70 ± 0.02	0.67 ± 0.03	1.31 ± 0.05
a99SBdisp	a99SBdisp-water	14.72 ± 0.02	15.29 ± 0.06	14.70 ± 0.02	14.09 ± 0.04	0.57 ± 0.05	-0.03 ± 0.01	-0.63 ± 0.04	0.59 ± 0.05	1.20 ± 0.09
ff99SBws	TIP4P/2005	14.75 ± 0.02	15.42 ± 0.05	14.72 ± 0.02	14.04 ± 0.05	0.66 ± 0.05	-0.04 ± 0.02	-0.71 ± 0.05	0.70 ± 0.05	1.37 ± 0.09
ff99SBws	TIP4P/2005s	14.78 ± 0.03	15.49 ± 0.05	14.73 ± 0.03	13.99 ± 0.04	0.71 ± 0.05	-0.05 ± 0.02	-0.79 ± 0.03	0.76 ± 0.04	1.50 ± 0.07

Table S3: R_g, ΔR_g, and ΔR_g^{SAS} values from unrestrained MD simulations of 18 different combinations of protein force fields and water models for the protein RNaseA.

force field	water model	R_g^{Prot} (Å)	R_g^{SAXS} (Å)	R_g^{SANS/H_2O} (Å)	R_g^{SANS/D_2O} (Å)	$R_g^{SAXS} - R_g^{Prot}$ (Å)	$R_g^{SANS/H_2O} - R_g^{Prot}$ (Å)	$R_g^{SANS/D_2O} - R_g^{Prot}$ (Å)	$R_g^{SAXS} - R_g^{SANS/H_2O}$ (Å)	$R_g^{SAXS} - R_g^{SANS/D_2O}$ (Å)
c36	SPC/E	15.53 ± 0.02	16.26 ± 0.05	15.61 ± 0.02	15.00 ± 0.03	0.73 ± 0.04	0.08 ± 0.01	-0.53 ± 0.03	0.65 ± 0.04	1.25 ± 0.07
ff14SB	SPC/E	15.31 ± 0.01	16.04 ± 0.03	15.39 ± 0.02	14.75 ± 0.03	0.73 ± 0.03	0.08 ± 0.01	-0.56 ± 0.02	0.65 ± 0.03	1.29 ± 0.05
ff99SB-ildn	SPC/E	15.37 ± 0.01	16.09 ± 0.03	15.44 ± 0.01	14.81 ± 0.02	0.72 ± 0.03	0.07 ± 0.01	-0.56 ± 0.02	0.65 ± 0.03	1.28 ± 0.04
c36	cTIP3P	15.62 ± 0.06	16.15 ± 0.03	15.70 ± 0.05	15.22 ± 0.08	0.52 ± 0.05	0.08 ± 0.02	-0.40 ± 0.03	0.45 ± 0.04	0.93 ± 0.07
c36	TIP3P	15.47 ± 0.04	16.04 ± 0.06	15.54 ± 0.04	15.04 ± 0.04	0.58 ± 0.04	0.08 ± 0.01	-0.43 ± 0.03	0.50 ± 0.04	1.01 ± 0.06
ff14SB	TIP3P	15.32 ± 0.01	16.02 ± 0.04	15.40 ± 0.01	14.78 ± 0.03	0.71 ± 0.05	0.08 ± 0.01	-0.54 ± 0.03	0.62 ± 0.04	1.24 ± 0.07
ff99SB-ildn	TIP3P	15.52 ± 0.02	16.30 ± 0.04	15.60 ± 0.02	14.95 ± 0.03	0.78 ± 0.04	0.08 ± 0.01	-0.57 ± 0.03	0.70 ± 0.04	1.35 ± 0.06
ff15fb	TIP3P-FB	15.33 ± 0.02	15.99 ± 0.04	15.39 ± 0.02	14.76 ± 0.03	0.66 ± 0.04	0.06 ± 0.01	-0.57 ± 0.03	0.60 ± 0.04	1.23 ± 0.06
c36	OPC3	15.50 ± 0.02	16.27 ± 0.04	15.58 ± 0.03	14.94 ± 0.03	0.76 ± 0.03	0.07 ± 0.01	-0.56 ± 0.02	0.69 ± 0.02	1.32 ± 0.04
ff99SBws	OPC3	15.57 ± 0.04	16.40 ± 0.04	15.65 ± 0.03	14.95 ± 0.05	0.82 ± 0.03	0.08 ± 0.01	-0.63 ± 0.03	0.75 ± 0.03	1.45 ± 0.06
c36	OPC	15.49 ± 0.02	16.10 ± 0.04	15.53 ± 0.02	15.01 ± 0.03	0.61 ± 0.03	0.04 ± 0.01	-0.49 ± 0.02	0.57 ± 0.03	1.09 ± 0.05
ff99SBws	OPC	15.43 ± 0.02	16.14 ± 0.03	15.51 ± 0.02	14.87 ± 0.03	0.71 ± 0.03	0.08 ± 0.01	-0.56 ± 0.02	0.63 ± 0.03	1.28 ± 0.04
ff99SBws	TIP4P-D	15.42 ± 0.02	16.24 ± 0.04	15.50 ± 0.02	14.79 ± 0.03	0.82 ± 0.03	0.08 ± 0.01	-0.63 ± 0.03	0.74 ± 0.03	1.45 ± 0.05
DES-amber	TIP4P-D	15.35 ± 0.02	16.20 ± 0.03	15.40 ± 0.02	14.63 ± 0.03	0.85 ± 0.02	0.05 ± 0.01	-0.72 ± 0.02	0.80 ± 0.02	1.57 ± 0.04
DES-amberSF1.0	TIP4P-D	15.51 ± 0.02	16.30 ± 0.04	15.52 ± 0.02	14.91 ± 0.04	0.79 ± 0.04	0.01 ± 0.02	-0.60 ± 0.03	0.78 ± 0.04	1.39 ± 0.07
a99SBdisp	a99SBdisp-water	15.65 ± 0.04	16.38 ± 0.07	15.71 ± 0.04	15.08 ± 0.04	0.73 ± 0.04	0.06 ± 0.01	-0.57 ± 0.03	0.67 ± 0.04	1.30 ± 0.06
ff99SBws	TIP4P/2005	15.41 ± 0.03	16.20 ± 0.05	15.45 ± 0.03	14.81 ± 0.02	0.80 ± 0.03	0.04 ± 0.01	-0.60 ± 0.02	0.76 ± 0.03	1.40 ± 0.04
ff99SBws	TIP4P/2005s	15.57 ± 0.03	16.52 ± 0.05	15.61 ± 0.03	14.81 ± 0.05	0.95 ± 0.04	0.04 ± 0.01	-0.75 ± 0.03	0.91 ± 0.04	1.70 ± 0.07

Table S4: R_g, ΔR_g, and ΔR_g^{SAS} values from unrestrained MD simulations of 18 different combinations of protein force fields and water models for the protein xylanase.

force field	water model	R_g^{Prot} (Å)	R_g^{SAXS} (Å)	R_g^{SANS/H_2O} (Å)	R_g^{SANS/D_2O} (Å)	$R_g^{SAXS} - R_g^{Prot}$ (Å)	$R_g^{SANS/H_2O} - R_g^{Prot}$ (Å)	$R_g^{SANS/D_2O} - R_g^{Prot}$ (Å)	$R_g^{SAXS} - R_g^{SANS/H_2O}$ (Å)	$R_g^{SAXS} - R_g^{SANS/D_2O}$ (Å)
c36	SPC/E	32.22 ± 0.01	34.00 ± 0.01	32.63 ± 0.01	31.48 ± 0.02	1.78 ± 0.02	0.41 ± 0.01	-0.75 ± 0.02	1.37 ± 0.02	2.52 ± 0.03
ff14SB	SPC/E	32.15 ± 0.01	33.74 ± 0.02	32.59 ± 0.01	31.53 ± 0.02	1.59 ± 0.03	0.44 ± 0.01	-0.63 ± 0.02	1.15 ± 0.02	2.21 ± 0.03
ff99SB-ildn	SPC/E	32.28 ± 0.01	33.85 ± 0.02	32.71 ± 0.01	31.67 ± 0.01	1.57 ± 0.02	0.43 ± 0.01	-0.61 ± 0.01	1.14 ± 0.02	2.18 ± 0.02
c36	cTIP3P	32.46 ± 0.02	34.08 ± 0.02	32.90 ± 0.02	31.82 ± 0.02	1.62 ± 0.01	0.43 ± 0.01	-0.64 ± 0.01	1.19 ± 0.02	2.26 ± 0.02
c36	TIP3P	32.26 ± 0.01	33.97 ± 0.02	32.67 ± 0.01	31.55 ± 0.02	1.71 ± 0.02	0.41 ± 0.01	-0.71 ± 0.02	1.30 ± 0.02	2.43 ± 0.03
ff14SB	TIP3P	32.22 ± 0.01	33.73 ± 0.02	32.66 ± 0.01	31.66 ± 0.02	1.51 ± 0.02	0.45 ± 0.01	-0.55 ± 0.02	1.07 ± 0.02	2.07 ± 0.03
ff99SB-ildn	TIP3P	32.39 ± 0.01	33.89 ± 0.01	32.84 ± 0.01	31.84 ± 0.02	1.49 ± 0.01	0.44 ± 0.01	-0.55 ± 0.02	1.05 ± 0.02	2.04 ± 0.02
ff15fb	TIP3P-FB	32.06 ± 0.01	33.54 ± 0.02	32.48 ± 0.01	31.44 ± 0.01	1.47 ± 0.02	0.42 ± 0.01	-0.62 ± 0.02	1.05 ± 0.02	2.10 ± 0.03
c36	OPC3	32.24 ± 0.01	33.93 ± 0.02	32.65 ± 0.01	31.57 ± 0.01	1.69 ± 0.02	0.42 ± 0.01	-0.67 ± 0.01	1.27 ± 0.02	2.36 ± 0.03
ff99SBws	OPC3	32.33 ± 0.01	33.93 ± 0.01	32.76 ± 0.01	31.70 ± 0.01	1.60 ± 0.01	0.44 ± 0.01	-0.62 ± 0.01	1.16 ± 0.01	2.22 ± 0.02
c36	OPC	32.30 ± 0.01	34.02 ± 0.03	32.71 ± 0.01	31.60 ± 0.02	1.72 ± 0.03	0.41 ± 0.01	-0.70 ± 0.02	1.31 ± 0.03	2.42 ± 0.04
ff99SBws	OPC	32.44 ± 0.02	34.00 ± 0.02	32.87 ± 0.02	31.82 ± 0.03	1.56 ± 0.02	0.43 ± 0.01	-0.62 ± 0.02	1.13 ± 0.02	2.18 ± 0.03
ff99SBws	TIP4P-D	32.42 ± 0.02	34.04 ± 0.02	32.85 ± 0.03	31.77 ± 0.03	1.62 ± 0.02	0.43 ± 0.01	-0.65 ± 0.01	1.19 ± 0.02	2.27 ± 0.03
DES-amber	TIP4P-D	32.30 ± 0.01	33.77 ± 0.02	32.72 ± 0.01	31.78 ± 0.02	1.47 ± 0.02	0.42 ± 0.01	-0.53 ± 0.01	1.05 ± 0.02	2.00 ± 0.03
DES-amberSF1.0	TIP4P-D	32.35 ± 0.01	33.93 ± 0.02	32.78 ± 0.02	31.74 ± 0.02	1.58 ± 0.02	0.43 ± 0.01	-0.60 ± 0.01	1.15 ± 0.02	2.19 ± 0.03
a99SBdisp	a99SBdisp-water	32.29 ± 0.02	33.83 ± 0.03	32.73 ± 0.02	31.66 ± 0.02	1.54 ± 0.02	0.44 ± 0.01	-0.62 ± 0.02	1.10 ± 0.02	2.17 ± 0.03
ff99SBws	TIP4P/2005	32.36 ± 0.02	34.01 ± 0.02	32.78 ± 0.02	31.69 ± 0.03	1.65 ± 0.02	0.43 ± 0.01	-0.66 ± 0.02	1.23 ± 0.02	2.32 ± 0.03
ff99SBws	TIP4P/2005s	32.50 ± 0.03	34.16 ± 0.04	32.94 ± 0.03	31.84 ± 0.02	1.66 ± 0.02	0.44 ± 0.01	-0.66 ± 0.01	1.21 ± 0.02	2.31 ± 0.03

Table S5: R_g, ΔR_g, and ΔR_g^{SAS} values from unrestrained MD simulations of 18 different combinations of protein force fields and water models for the protein glucose isomerase.

force field	water model	R_g^{Prot} (Å)	R_g^{SAXS} (Å)	R_g^{SANS/H_2O} (Å)	R_g^{SANS/D_2O} (Å)	$R_g^{SAXS} - R_g^{Prot}$ (Å)	$R_g^{SANS/H_2O} - R_g^{Prot}$ (Å)	$R_g^{SANS/D_2O} - R_g^{Prot}$ (Å)	$R_g^{SAXS} - R_g^{SANS/H_2O}$ (Å)	$R_g^{SAXS} - R_g^{SANS/D_2O}$ (Å)
c36	SPC/E	14.17 ± 0.02	14.95 ± 0.04	14.32 ± 0.03	13.11 ± 0.04	0.78 ± 0.05	0.15 ± 0.02	-1.06 ± 0.03	0.63 ± 0.05	1.84 ± 0.07
ff14SB	SPC/E	14.13 ± 0.01	14.78 ± 0.04	14.25 ± 0.02	13.11 ± 0.04	0.65 ± 0.04	0.12 ± 0.02	-1.02 ± 0.04	0.53 ± 0.04	1.66 ± 0.07
ff99SB-ildn	SPC/E	14.06 ± 0.01	14.82 ± 0.04	14.18 ± 0.01	12.99 ± 0.04	0.76 ± 0.04	0.12 ± 0.01	-1.07 ± 0.04	0.64 ± 0.05	1.83 ± 0.08
c36	cTIP3P	14.31 ± 0.02	14.98 ± 0.03	14.43 ± 0.02	13.31 ± 0.03	0.66 ± 0.03	0.12 ± 0.01	-1.00 ± 0.03	0.54 ± 0.03	1.66 ± 0.06
c36	TIP3P	14.08 ± 0.02	14.80 ± 0.05	14.22 ± 0.03	13.11 ± 0.04	0.72 ± 0.04	0.14 ± 0.02	-0.97 ± 0.04	0.58 ± 0.05	1.69 ± 0.07
ff14SB	TIP3P	14.12 ± 0.01	14.86 ± 0.04	14.25 ± 0.01	13.10 ± 0.03	0.74 ± 0.04	0.12 ± 0.01	-1.02 ± 0.03	0.62 ± 0.04	1.76 ± 0.06
ff99SB-ildn	TIP3P	14.06 ± 0.02	14.84 ± 0.03	14.19 ± 0.03	13.02 ± 0.02	0.78 ± 0.03	0.12 ± 0.01	-1.04 ± 0.02	0.65 ± 0.02	1.82 ± 0.03
ff15fb	TIP3P-FB	14.02 ± 0.01	14.73 ± 0.05	14.14 ± 0.02	12.90 ± 0.04	0.71 ± 0.05	0.12 ± 0.02	-1.12 ± 0.04	0.59 ± 0.04	1.83 ± 0.08
c36	OPC3	14.13 ± 0.02	14.92 ± 0.04	14.25 ± 0.03	13.02 ± 0.05	0.78 ± 0.04	0.12 ± 0.02	-1.11 ± 0.04	0.67 ± 0.05	1.89 ± 0.07
ff99SBws	OPC3	14.01 ± 0.02	14.76 ± 0.04	14.11 ± 0.02	12.93 ± 0.04	0.76 ± 0.04	0.10 ± 0.01	-1.08 ± 0.03	0.65 ± 0.03	1.84 ± 0.06
c36	OPC	14.30 ± 0.03	14.94 ± 0.06	14.41 ± 0.03	13.29 ± 0.04	0.63 ± 0.05	0.11 ± 0.02	-1.01 ± 0.05	0.52 ± 0.05	1.64 ± 0.09
ff99SBws	OPC	14.12 ± 0.01	14.82 ± 0.05	14.23 ± 0.02	13.06 ± 0.04	0.69 ± 0.05	0.10 ± 0.02	-1.07 ± 0.04	0.59 ± 0.05	1.76 ± 0.09
ff99SBws	TIP4P-D	14.12 ± 0.02	14.84 ± 0.03	14.26 ± 0.02	13.05 ± 0.03	0.72 ± 0.03	0.14 ± 0.01	-1.07 ± 0.03	0.58 ± 0.03	1.79 ± 0.06
DES-amber	TIP4P-D	14.25 ± 0.01	15.02 ± 0.04	14.37 ± 0.01	13.14 ± 0.03	0.77 ± 0.04	0.12 ± 0.01	-1.12 ± 0.03	0.65 ± 0.04	1.89 ± 0.07
DES-amberSF1.0	TIP4P-D	14.22 ± 0.02	15.06 ± 0.04	14.31 ± 0.02	12.99 ± 0.03	0.84 ± 0.04	0.10 ± 0.02	-1.23 ± 0.03	0.74 ± 0.04	2.07 ± 0.06
a99SBdisp	a99SBdisp-water	14.10 ± 0.03	14.82 ± 0.06	14.24 ± 0.03	13.07 ± 0.04	0.71 ± 0.05	0.14 ± 0.01	-1.04 ± 0.04	0.57 ± 0.05	1.75 ± 0.08
ff99SBws	TIP4P/2005	13.97 ± 0.02	14.75 ± 0.04	14.09 ± 0.03	12.97 ± 0.03	0.78 ± 0.03	0.12 ± 0.01	-1.00 ± 0.04	0.66 ± 0.03	1.78 ± 0.06
ff99SBws	TIP4P/2005s	14.03 ± 0.03	14.83 ± 0.05	14.13 ± 0.03	12.91 ± 0.04	0.80 ± 0.04	0.10 ± 0.01	-1.12 ± 0.04	0.70 ± 0.04	1.92 ± 0.07

Table S6: R_g, ΔR_g, and ΔR_g^{SAS} values from unrestrained MD simulations of 18 different combinations of protein force fields and water models for the protein lysozyme.

force field	water model	R_g^{Prot} (Å)	R_g^{SAXS} (Å)	R_g^{SANS/H_2O} (Å)	R_g^{SANS/D_2O} (Å)	$R_g^{SAXS} - R_g^{Prot}$ (Å)	$R_g^{SANS/H_2O} - R_g^{Prot}$ (Å)	$R_g^{SANS/D_2O} - R_g^{Prot}$ (Å)	$R_g^{SAXS} - R_g^{SANS/H_2O}$ (Å)	$R_g^{SAXS} - R_g^{SANS/D_2O}$ (Å)
c36	SPC/E	31.19 ± 0.01	32.15 ± 0.02	31.57 ± 0.01	31.22 ± 0.02	0.96 ± 0.02	0.38 ± 0.01	0.03 ± 0.02	0.58 ± 0.02	0.92 ± 0.03
ff14SB	SPC/E	31.27 ± 0.02	32.28 ± 0.03	31.65 ± 0.01	31.25 ± 0.02	1.02 ± 0.03	0.38 ± 0.01	-0.02 ± 0.02	0.63 ± 0.03	1.04 ± 0.04
ff99SB-ildn	SPC/E	31.50 ± 0.02	32.58 ± 0.03	31.88 ± 0.02	31.46 ± 0.02	1.08 ± 0.02	0.37 ± 0.01	-0.05 ± 0.01	0.70 ± 0.02	1.12 ± 0.03
c36	cTIP3P	31.30 ± 0.02	32.14 ± 0.05	31.65 ± 0.02	31.39 ± 0.03	0.84 ± 0.04	0.35 ± 0.01	0.09 ± 0.02	0.49 ± 0.04	0.75 ± 0.05
c36	TIP3P	31.23 ± 0.02	32.10 ± 0.02	31.59 ± 0.01	31.30 ± 0.02	0.87 ± 0.02	0.36 ± 0.01	0.07 ± 0.01	0.50 ± 0.02	0.80 ± 0.02
ff14SB	TIP3P	31.16 ± 0.02	32.14 ± 0.02	31.55 ± 0.02	31.16 ± 0.03	0.99 ± 0.02	0.39 ± 0.01	-0.00 ± 0.01	0.60 ± 0.02	0.99 ± 0.02
ff99SB-ildn	TIP3P	31.31 ± 0.01	32.30 ± 0.02	31.69 ± 0.01	31.31 ± 0.02	0.99 ± 0.02	0.39 ± 0.01	0.01 ± 0.02	0.60 ± 0.02	0.98 ± 0.03
ff15fb	TIP3P-FB	31.10 ± 0.02	32.01 ± 0.02	31.48 ± 0.02	31.11 ± 0.02	0.91 ± 0.02	0.38 ± 0.01	0.01 ± 0.01	0.53 ± 0.02	0.90 ± 0.02
c36	OPC3	31.21 ± 0.03	32.21 ± 0.05	31.58 ± 0.03	31.20 ± 0.03	1.00 ± 0.03	0.37 ± 0.01	-0.01 ± 0.02	0.63 ± 0.03	1.01 ± 0.04
ff99SBws	OPC3	31.49 ± 0.02	32.56 ± 0.02	31.86 ± 0.02	31.45 ± 0.03	1.07 ± 0.02	0.37 ± 0.01	-0.04 ± 0.02	0.70 ± 0.02	1.11 ± 0.03
c36	OPC	31.35 ± 0.02	32.28 ± 0.03	31.69 ± 0.02	31.39 ± 0.03	0.93 ± 0.02	0.34 ± 0.01	0.04 ± 0.02	0.59 ± 0.02	0.90 ± 0.03
ff99SBws	OPC	31.63 ± 0.03	32.63 ± 0.05	31.97 ± 0.02	31.64 ± 0.03	1.00 ± 0.04	0.35 ± 0.01	0.01 ± 0.02	0.65 ± 0.04	0.99 ± 0.05
ff99SBws	TIP4P-D	31.54 ± 0.02	32.56 ± 0.02	31.93 ± 0.02	31.54 ± 0.02	1.02 ± 0.02	0.39 ± 0.01	-0.00 ± 0.01	0.63 ± 0.02	1.02 ± 0.03
DES-amber	TIP4P-D	31.35 ± 0.01	32.28 ± 0.03	31.70 ± 0.02	31.39 ± 0.02	0.94 ± 0.02	0.35 ± 0.01	0.04 ± 0.01	0.58 ± 0.02	0.89 ± 0.03
DES-amberSF1.0	TIP4P-D	31.32 ± 0.02	32.40 ± 0.03	31.68 ± 0.02	31.26 ± 0.03	1.08 ± 0.02	0.36 ± 0.01	-0.06 ± 0.01	0.72 ± 0.02	1.14 ± 0.03
a99SBdisp	a99SBdisp-water	31.38 ± 0.02	32.31 ± 0.03	31.76 ± 0.01	31.42 ± 0.01	0.93 ± 0.02	0.38 ± 0.01	0.03 ± 0.02	0.55 ± 0.03	0.89 ± 0.04
ff99SBws	TIP4P/2005	31.47 ± 0.01	32.50 ± 0.02	31.84 ± 0.01	31.44 ± 0.01	1.03 ± 0.02	0.37 ± 0.01	-0.03 ± 0.01	0.66 ± 0.02	1.06 ± 0.03
ff99SBws	TIP4P/2005s	31.57 ± 0.01	32.66 ± 0.02	31.94 ± 0.02	31.48 ± 0.02	1.09 ± 0.02	0.37 ± 0.01	-0.09 ± 0.02	0.72 ± 0.03	1.18 ± 0.04

Table S7: R_g, ΔR_g, and ΔR_g^{SAS} values from unrestrained MD simulations of 18 different combinations of protein force fields and water models for the protein urate oxidase.

Supplementary References

(1) Trewhella, J.; Vachette, P.; Bierma, J.; Blanchet, C.; Brookes, E.; Chakravarthy, S.; Chatzimagas, L.; Cleveland, T.; Cowieson, N.; Crossett, B. e. a. A Round Robin Approach Provides a Detailed Assessment of Biomolecular Small-Angle Scattering Data Reproducibility and Yields Consensus Curves for Benchmarking. *Acta Cryst. D78* **2022**,

(2) Svergun, D. I.; Richard, S.; Koch, M. H. J.; Sayers, Z.; Kuprin, S.; Zaccai, G. Protein hydration in solution: Experimental observation by x-ray and neutron scattering. *Proc. Natl. Acad. Sci. U.S.A.* **1998**, *95*, 2267–2272.

(3) Grishaev, A.; Guo, L.; Irving, T.; Bax, A. Improved Fitting of Solution X-ray Scattering Data to Macromolecular Structures and Structural Ensembles by Explicit Water Modeling. *J. Am. Chem. Soc.* **2010**, *132*, 15484–15486.

(4) Ivanović, M. T.; Bruetzel, L. K.; Shevchuk, R.; Lipfert, J.; Hub, J. S. Quantifying the influence of the ion cloud on SAXS profiles of charged proteins. *Phys. Chem. Chem. Phys.* **2018**, *20*, 26351–26361.

(5) Holst, M. J. The Poisson-Boltzmann Equation: Analysis and Multilevel Numerical Solution. Ph.D. thesis, California institute of Technology, 1994.

(6) Venable, R. M.; Luo, Y.; Gawrisch, K.; Roux, B.; Pastor, R. W. Simulations of Anionic Lipid Membranes: Development of Interaction-Specific Ion Parameters and Validation Using NMR Data. *J. Phys. Chem. B* **2013**, *117*, 10183–10192.

(7) Yoo, J.; Aksimentiev, A. New Tricks for Old Dogs: Improving the Accuracy of Biomolecular Force Fields by Pair-Specific Corrections to Non-Bonded Interactions. *Phys. Chem. Chem. Phys.* **2018**, *20*, 8432–8449.

(8) Ivanović, M. T.; Bruetzel, L. K.; Shevchuk, R.; Lipfert, J.; Hub, J. S. Quantifying the Influence of the Ion Cloud on SAXS Profiles of Charged Proteins. *Physical Chemistry Chemical Physics* **2018**, *20*, 26351–26361.

(9) Chen, P.-c.; Hub, J. S. Validating Solution Ensembles from Molecular Dynamics Simulation by Wide-Angle X-ray Scattering Data. *Biophys. J.* **2014**, *107*, 435–447.

(10) Chatzimagas, L.; Hub, J. S. In *Small Angle Scattering Part A: Methods for Structural Investigation*; Tainer, J. A., Ed.; Methods in Enzymology; Academic Press, 2022; Vol. 677; pp 433–456.

(11) Merzel, F.; Smith, J. C. Is the First Hydration Shell of Lysozyme of Higher Density than Bulk Water? *Proc. Natl. Acad. Sci. USA* **2002**, *99*, 5378–5383.

(12) Chen, P.-c.; Hub, J. Validating Solution Ensembles from Molecular Dynamics Simulation by Wide-Angle X-ray Scattering Data. *Biophys. J.* **2014**, *107*, 435–447.

(13) Wang, J.; Dauter, M.; Alkire, R.; Joachimiak, A.; Dauter, Z. Triclinic lysozyme at 0.65 Å resolution. *Acta Cryst. D* **2007**, *63*, 1254–1268.

(14) Wlodawer, A.; Svensson, L. A.; Sjoelin, L.; Gilliland, G. L. Structure of phosphate-free ribonuclease A refined at 1.26 Å. *Biochem.* **1988**, *27*, 2705–2717.

(15) Watanabe, N.; Akiba, T.; Kanai, R.; Harata, K. Structure of an orthorhombic form of xylanase II from *Trichoderma reesei* and analysis of thermal displacement. *Acta Cryst. D* **2006**, *62*, 784–792.

(16) Nowak, E.; Panjikar, S.; Tucker, P. Atomic structure of Glucose isomerase . *To be published*

(17) Gabison, L.; Chiadmi, M.; El Hajji, M.; Castro, B.; Colloc'h, N.; Prangé, T. Near-atomic resolution structures of urate oxidase complexed with its substrate and analogues: the protonation state of the ligand. *Acta Cryst. D* **2010**, *66*, 714–724.

(18) Huang, J.; Rauscher, S.; Nawrocki, G.; Ting, R.; Feig, M.; de Groot, B.; Grubmüller, H.; MacKerell, A. CHARMM36m: An Improved Force Field for Folded and Intrinsically Disordered Proteins. *Nat. Methods* **2017**, *14*, 71–73.

(19) MacKerell, A. D. J. et al. All-Atom Empirical Potential for Molecular Modeling and Dynamics Studies of Proteins. *J. Phys. Chem. B* **1998**, *102*, 3586–3616.

(20) Jorgensen, W. L.; Chandrasekhar, J.; Madura, J. D.; Impey, R. W.; Klein, M. L. Comparison of simple potential functions for simulating liquid water. *J. Chem. Phys.* **1983**, *79*, 926–935.

(21) Berendsen, H. J. C.; Grigera, J. R.; Straatsma, T. P. The missing term in effective pair potentials. *J. Phys. Chem.* **1987**, *91*, 6269–6271.

(22) Izadi, S.; Onufriev, A. V. Accuracy limit of rigid 3-point water models. *J. Chem. Phys.* **2016**, *145*, 074501.

(23) Izadi, S.; Anandakrishnan, R.; Onufriev, A. V. Building Water Models: A Different Approach. *J. Phys. Chem. Lett.* **2014**, *5*, 3863–3871.

(24) Maier, J. A.; Martinez, C.; Kasavajhala, K.; Wickstrom, L.; Hauser, K. E.; Simmerling, C. ff14SB: Improving the Accuracy of Protein Side Chain and Backbone Parameters from ff99SB. *J. Chem. Theory Comput.* **2015**, *11*, 3696–3713.

(25) Lindorff-Larsen, K.; Piana, S.; Palmo, K.; Maragakis, P.; Klepeis, J. L.; Dror, R. O.; Shaw, D. E. Improved side-chain torsion potentials for the Amber ff99SB protein force field. *Proteins* **2010**, *78*, 1950–1958.

(26) Wang, L.-P.; McKiernan, K. A.; Gomes, J.; Beauchamp, K. A.; Head-Gordon, T.; Rice, J. E.; Swope, W. C.; Martínez, T. J.; Pande, V. S. Building a More Predictive Protein Force Field: A Systematic and Reproducible Route to AMBER-FB15. *J. Phys. Chem. B* **2017**, *121*, 4023–4039.

(27) Best, R. B.; Hummer, G. Optimized Molecular Dynamics Force Fields Applied to the Helix-Coil Transition of Polypeptides. *J. Phys. Chem. B* **2009**, *113*, 9004–9015.

(28) Best, R. B.; Zheng, W.; Mittal, J. Balanced Protein–Water Interactions Improve Properties of Disordered Proteins and Non-Specific Protein Association. *J. Chem. Theory Comput.* **2014**, *10*, 5113–5124.

(29) Abascal, J. L. F.; Vega, C. A general purpose model for the condensed phases of water: TIP4P/2005. *J. Chem. Phys.* **2005**, *123*, 234505.

(30) Piana, S.; Donchev, A. G.; Robustelli, P.; Shaw, D. E. Water Dispersion Interactions Strongly Influence Simulated Structural Properties of Disordered Protein States. *J. Phys. Chem. B* **2015**, *119*, 5113–5123.

(31) Piana, S.; Robustelli, P.; Tan, D.; Chen, S.; Shaw, D. E. Development of a Force Field for the Simulation of Single-Chain Proteins and Protein–Protein Complexes. *J. Chem. Theory Comput.* **2020**, *16*, 2494–2507.

(32) Robustelli, P.; Piana, S.; Shaw, D. E. Developing a molecular dynamics force field for both folded and disordered protein states. *Proc. Natl. Acad. Sci. U.S.A.* **2018**, *115*, E4758–E4766.
