# Supplementary material for: Scrutinizing the protein hydration shell from molecular dynamics simulations against consensus small-angle scattering data
Source: Commun Chem. 2023 Dec 12;6:272. doi: 10.1038/s42004-023-01067-1 (PMC10716392; doi:10.1038/s42004-023-01067-1)
Supplement: Supplementary file 3 — Description of Additional Supplementary File [file 42004_2023_1067_MOESM3_ESM.pdf]

Description of additional supplementary information

Johanna-Barbara Linse and Jochen S. Hub*

Theoretical Physics and Center for Biophysics, Saarland University, Saarbrücken, 66123, Germany

E-mail: jochen.hub@uni-saarland.de

Supplementary Movie 1: Illustration of the hydration layers around the protein xylanase.

Three-dimensional solvent electron density taken from a simulation of xylanase obtained with ff99SBws and TIP4P/2005s water using position restraints on all heavy atoms. Only density inside the envelope is shown in shades from light grey (bulk water) to blue to orange, revealing the first (orange) and the second (mostly blue) hydration layers. For the color code, see the color bar in Fig. 1B.
